# Radiochemistry for positron emission tomography

**DOI:** 10.1038/s41467-023-36377-4

**Published:** 2023-06-05

**Authors:** Jian Rong, Ahmed Haider, Troels E. Jeppesen, Lee Josephson, Steven H. Liang

**Affiliations:** 1 Department of Radiology and Imaging Sciences, Emory University, 1364 Clifton Rd, Atlanta, GA 30322 USA; 2 Division of Nuclear Medicine and Molecular Imaging, Massachusetts General Hospital & Department of Radiology, Harvard Medical School, Boston, MA 02114 USA

**Keywords:** Medicinal chemistry, Radionuclide imaging

## Abstract

Positron emission tomography (PET) constitutes a functional imaging technique that is harnessed to probe biological processes in vivo. PET imaging has been used to diagnose and monitor the progression of diseases, as well as to facilitate drug development efforts at both preclinical and clinical stages. The wide applications and rapid development of PET have ultimately led to an increasing demand for new methods in radiochemistry, with the aim to expand the scope of synthons amenable for radiolabeling. In this work, we provide an overview of commonly used chemical transformations for the syntheses of PET tracers in all aspects of radiochemistry, thereby highlighting recent breakthrough discoveries and contemporary challenges in the field. We discuss the use of biologicals for PET imaging and highlight general examples of successful probe discoveries for molecular imaging with PET – with a particular focus on translational and scalable radiochemistry concepts that have been entered to clinical use.

## Abstract (web summary)

Positron emission tomography is widely used to diagnose and monitor different disease states and interest in the technique has led to the demand for the development of new method for radiolabelling. Here the authors review the recent progress in the development of new PET probes.

## Introduction

Translational molecular imaging has facilitated the diagnosis and management of numerous pathologies and has become an integral part of daily clinical routine—with significant implications in oncology, cardiology, and neurology^1–3^. In contrast to structural and anatomical imaging, molecular imaging provides functional information via the interaction between a targeted probe and the biological system. In nuclear medicine, the molecular probe constitutes a radionuclide-bearing radiopharmaceutical, frequently referred to as a tracer. From an imaging point of view, the field of nuclear medicine is composed of two mechanistically distinct, albeit somewhat comparable imaging techniques, namely positron emission tomography (PET) and single-photon emission computed tomography (SPECT). While the former imaging modality exploits coincidence detection of colinear annihilation photons, the latter is based on the detection of single gamma rays. Accordingly, although PET and SPECT probes are both generated via the incorporation of a radionuclide within a biomolecule, PET radionuclides emit positrons while SPECT radionuclides are γ-emitters. PET and SPECT are non-invasive techniques that provide information on a molecular and cellular level, which makes them particularly valuable for the detection of biochemical changes at an early stage of disease development, often before clinical symptoms are evident^4^.

### Positron Emission Tomography (PET)

**Fig. 1:**
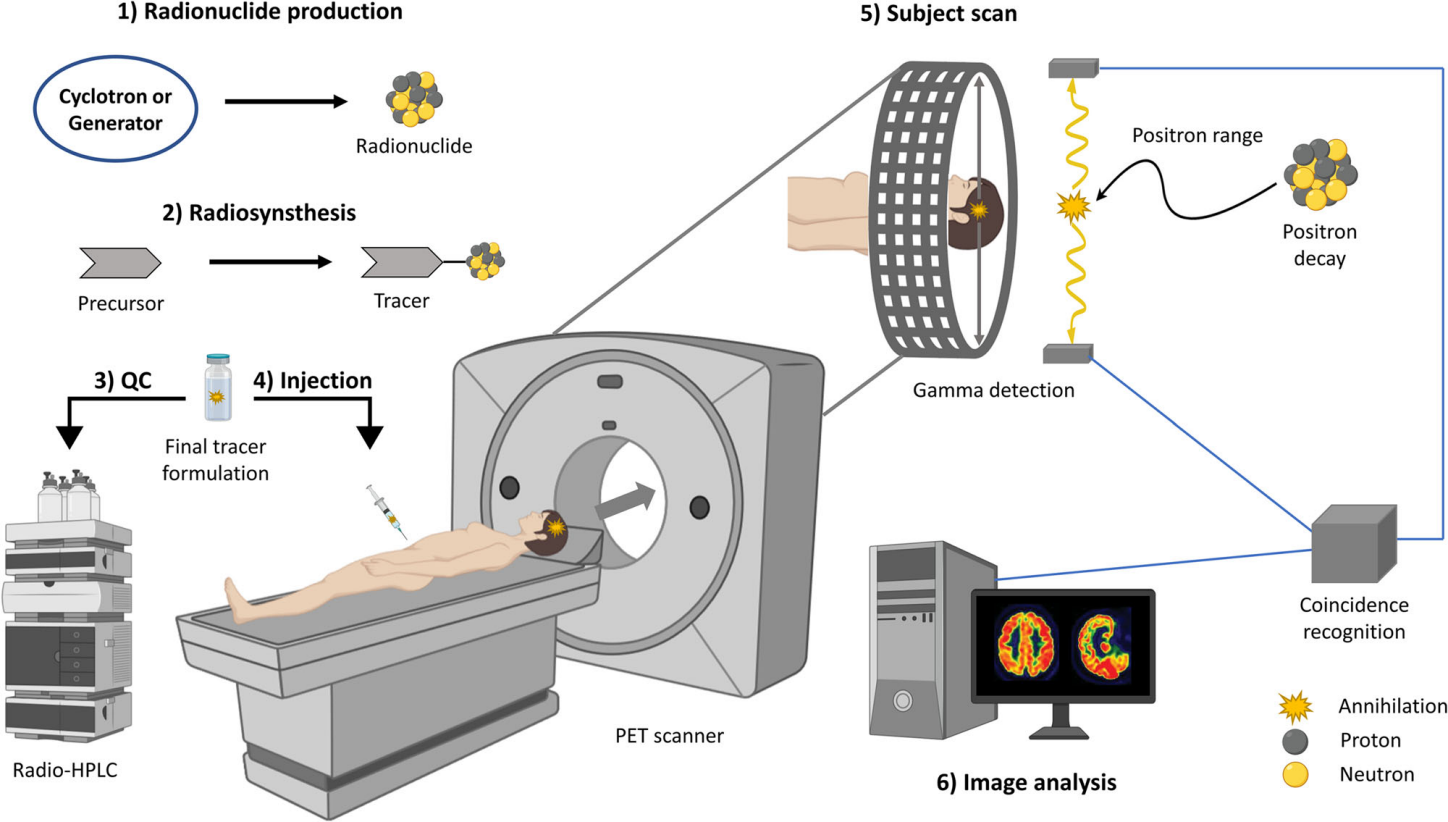
Principle of positron emission tomography (PET) imaging. 1) Radionuclide generation. 2) Tracer synthesis. 3) Quality control (QC). 4) Intravenous tracer injection. 5) PET scan: positron decay, annihilation, and coincidence detection. 6) Image analysis and data quantification. Created with BioRender.com.

Given the excellent sensitivity and the recent technical advances made in accurate image quantification, PET has been widely used for disease staging and drug development purposes^4–7^. Notably, only trace amounts of the radioactive probe are typically necessary to obtain high-quality images. Due to this remarkable sensitivity, PET radioligands have the capability to probe biological processes without eliciting pharmacological activity^8^. The principle of PET imaging is illustrated in Fig. 1. In the initial step, PET radionuclides are typically generated via nuclear reactions within a cyclotron or by specific decay mechanisms from a generator. The resulting radionuclides are incorporated through a variety of available radiochemical reactions into molecules, thus yielding the desired tracers. Quality control (QC) measures, according to legal requirements and monographs in the respective Pharmacopoeias (e.g., US and European Pharmacopoeia), are undertaken to ensure proper radiopharmaceutical quality for intravenous injection, before the tracer is administered to the patient. The patient is subsequently scanned in a round-shaped scanner, where quantification of the radioactive signal provides information on the annihilation site, and thereby, the tracer localization at different organs. Importantly, the emitted positron travels a certain distance (positron range), depending on its energy, and undergoes inelastic collisions until the kinetic energy is so low that the timely overlap with an electron and annihilation is possible. Annihilation typically converts the combined mass into electromagnetic energy that can be in the form of two gamma rays released in opposite directions. Spatial image resolution is inherently limited by the positron range of a given PET radionuclide. Coincidences of photons are detected by the full-ring array of detectors of the PET camera and annihilation sources are reconstructed. Indeed, through distinct algorithms, PET data can be reconstructed into the spatial distribution of a given probe. Major challenges in contemporary PET image reconstruction include the correction for scattering and random coincidence events as well as distinct signal attenuation through different body tissues. As such, PET imaging is routinely combined with computed tomography for anatomical orientation and attenuation correction^9^.

Among the various existing positron-emitting nuclides (Table 1), fluorine-18 (^18^F) is the most widely used radionuclide for PET due to its unique advantages over other PET nuclides. These advantages include (1) the low positron range of <1 mm^10^, which allows the generation of high spatial resolution images, (2) the clear positron emission profile comprising 97% positron emission and 3% electron capture (EC), and (3) the optimal physical half-live of 109.8 min that enables off-site use in satellite facilities without a cyclotron^11^. Notwithstanding the favorable physical properties of ^18^F, fluorine atom is typically not a constituent of most biomolecules and hence, frequently introduced via bioisosteric substitution of a hydrogen atom or hydroxyl functionality^5^. These substitutions are not associated with significant steric effects; however, electron-withdrawing fluorine atoms may substantially affect pharmacokinetic and pharmacodynamic properties of PET radioligands^12,13^. Given that carbon atoms are encountered in virtually every biomolecule, carbon-11 (^11^C) labeling is frequently considered a useful alternative to circumvent the need for bioisosteric substitution, despite the relatively short half-life and higher positron range as compared to ^18^F. Indeed, remarkable advances in ^11^C-labeling strategies have enabled the labeling of several functional groups, thereby substantially expanding the chemical space of substrates amenable to PET. Besides fluorine-18 and carbon-11, there is a number of other commonly used PET radionuclides (Table 1). In this review, we will summarize commonly used radiolabeling strategies in PET radiochemistry, thereby focusing on recent advances^8,14^ with fluorine-18, carbon-11, nitrogen-13, oxygen-15, and other PET radiohalogens, as well as chelator-based radiometal chemistry. Breakthrough examples of PET ligands with clinical relevance will be discussed. Further, we will elucidate how advances in bioconjugation methods in radiochemistry led to novel opportunities for the use of biologicals in molecular imaging with PET.

**Table 1:** Characteristics of commonly used positron emitting radionuclides^5,171,264–267^

Isotope	Half-life	Production^a^	Mode of decay	Common source
^18^F	109.8 min	^18^O(p,n)^18^F	β^+^ (97%), EC (3%)	Cyclotron
^11^C	20.4 min	^14^N(p,α)^11^C	β^+^ (100%)	Cyclotron
^13^N	10 min	^16^O(p,α)^13^N	β^+^ (100%)	Cyclotron
^15^O	2 min	^15^N(p,n)^15^O	β^+^ (100%)	Cyclotron
^124^I	4.2 d	^124^Te(p,n)^124^I	β^+^ (23%), EC (77%)	Cyclotron
^44^Sc	4.0 h	^44^Ti/^44^Sc	β^+^ (94%), EC (6%)	Generator^b^
^64^Cu	12.7 h	^64^Ni(p,n)^64^Cu	β^+^ (17%), EC (44%), β^-^ (39%)	Cyclotron^c^
^68^Ga	67.7 min	^68^Ge/^68^Ga	β^+^ (89%), EC (11%)	Generator^d^
^82^Rb	1.3 min	^82^Sr/^82^Rb	β^+^ (95%), EC (5%)	Generator
^86^Y	14.7 h	^86^Sr(p,n)^86^Y	β^+^ (32%), EC (68%)	Cyclotron
^89^Zr	78.4 h	^89^Y(p,n)^89^Zr	β^+^ (23%), EC (77%)	Cyclotron

^a^Nuclear reaction or decay mechanism. Data source: nndc.bnl.gov.

^b44^Sc can also be produced via ^nat^Ca(p,n)^44^Sc^268^ or ^47^Ti(p,α)^44^Sc^269^ using a cyclotron.

^c64^Cu can also be produced via ^63^Cu(n,γ)^64^Cu using a reactor^270^.

^d68^Ga can also be produced from zinc-68 using a cyclotron^271,272^.

### PET tracers as diagnostic biomarker and PK/PD tool in drug discovery

Over the past decades, PET imaging has substantially gained attention and is increasingly used not only as a preclinical and clinical research tool but also in routine clinical diagnosis. As such, PET is ideally suited for visualizing molecular and cellular events that occur early in the course of a disease or following therapeutic intervention^9^. Further, depending on the applied tracer, PET can be used to gain prognostic information, offering a valuable risk-stratification tool in clinical practice, as it is currently performed with various probes in atherosclerotic inflammation imaging^2,15^.

In addition to its role in disease diagnosis and therapy monitoring, PET has been established as a vital tool that is being used at various stages of modern drug development. As such, PET can be applied for target validation studies by assessing the target protein expression and localization at different disease stages^6^. Further, lead compound optimization is typically conducted by in vivo assessment of pharmacokinetic and pharmacodynamic drug properties. Proof of target engagement, brain penetration, and potential differences between patients and healthy individuals are of particular interest in PET studies. If amenable to labeling with a PET nuclide, drug candidates can be directly radiolabeled and injected into living organisms, thereby yielding information on drug pharmacokinetics and metabolic stability through blood sampling or post-mortem analysis. This approach is limited by the costly, and in some cases challenging, development of a suitable radiosynthesis for every drug candidate to be tested. As an alternative, promising compounds in development can be challenged by competitive target engagement studies against an established PET probe that shares the same binding site. The advantage of the latter method is that multiple compounds can be effectively screened against one single probe, thereby addressing the following questions: (1) Were the right patients selected to test a given hypothesis and which patient populations are most likely to benefit from this trial (patient stratification)? In many cases, obtaining histological evidence from biopsies is not feasible, whereas a non-invasive assessment of target abundancy in the organ of interest by PET may substantially improve patient selection. (2) Does the drug reach the desired target site and what exposure can be achieved (target occupancy at a given dose)? In addition, while histological assessment may be hampered by protein degradation outside of the human body, and only covers a limited area of the target tissue, PET provides real-time information, allowing whole-body imaging. Obtaining such information at early stage is of paramount value to improving clinical trials and establishing an efficient drug development process. Other applications include the use of PET as accompanying tests for precision therapies, whereas PET probes are frequently employed as basic and clinical research tools to study disease pathophysiology. Indeed, considering the quantitative nature of PET, non-invasive studies to elucidate disease mechanisms and pathophysiology are feasible not only in animal models but also in human subjects, as shown by various recent clinical studies^16–19^. The translational value of PET is further corroborated by the ability to directly monitor disease progression on a molecular level, thereby providing a unique functional readout that is complementary to structural and morphological information typically detected by conventional imaging modalities.

### Translational Radiochemistry

The successful clinical translation of a PET tracer depends not only on its biological characteristics but also on the implementation of practical radiolabeling solutions that allow fully automated tracer synthesis under required quality assurance levels (e.g., GMP, cGRPP). Indeed, the selection of PET nuclide and radiosynthetic approach merits careful considerations since synthetic conditions deemed appropriate for research applications may not be suitable to deliver pharmaceutical-grade tracers. Potential issues may arise from the lack of precursor stability at ambient temperature, the use of metal catalysts that may exert toxicity if injected into humans, synthetic steps that require manual intervention, volatile solvents with volume reproducibility issues, radioactive and non-radioactive byproducts that co-elute with the tracer, lengthy multistep syntheses that do not yield sufficient radioactivity or insufficient molar radioactivity levels at the end of synthesis, and the lack of tracer stability in the final formulation. The concept *translational radiochemistry* is introduced herein to emphasize the unique characteristics of PET chemistry aimed at human translation and routine radiopharmaceutical production. To qualify any novel radiochemical method from benchtop discovery as translational radiochemistry, we evaluate it not only by its radiochemical yield and molar activity, but also by the feasibility and translatability of automation or remotely-controlled radiosynthesis in a hot cell, reproducibility and regulatory compliance in a clinical production/manufacturing environment (e.g., cGMP process) with the operation, participation, and approval of a diverse team, including cyclotron engineers, radiochemists, QC/QA analysts, formulation specialists, radiopharmacists, and nuclear radiologists. As such, translational radiochemistry is refined as scalable, robust, and reproducible PET chemistry that consistently provides high-quality radiopharmaceuticals, meeting a predefined set of specifications for human use, which is critical to protect patients from unnecessary toxicities and ensure the best possible diagnostic care.

### PET chemistry of carbon-11, nitrogen-13 and oxygen-15

#### Carbon-11

**Fig. 2:**
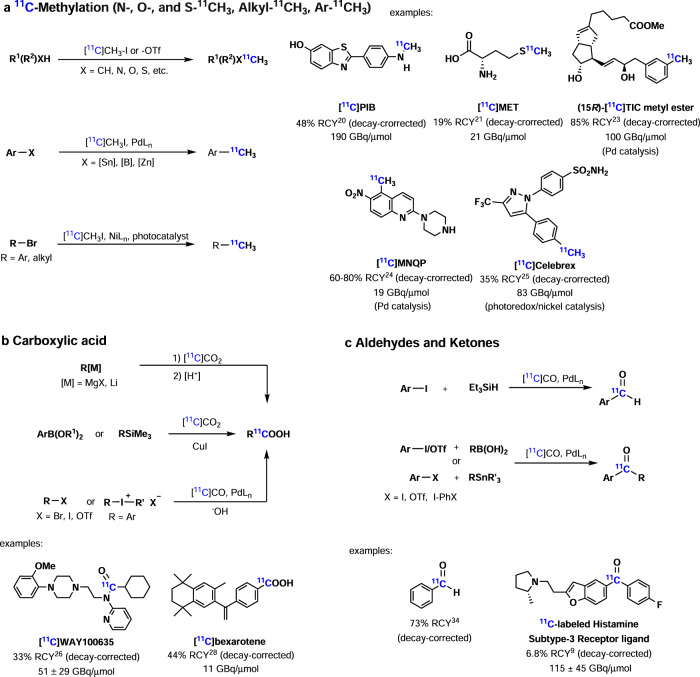
Carbon-11 chemistry. **a** ^11^C-methylation with [^11^C]CH_3_I or [^11^C]CH_3_OTf (*N*-, *O*-, *S*-, *C*-^11^C-methylation). **b** Synthesis of [^11^C]carboxylic acids. **c** Synthesis of [^11^C]aldehydes and [^11^C]ketones.

Given that carbon atoms are encountered in the vast majority of biologically active molecules, increasing efforts have been devoted to advancing carbon-11 (^11^C, half-life *t*_1/2_ = 20 min) labeling strategies, thus yielding a plethora of [^11^C]methyl-, [^11^C]carbonyl-, [^11^C]cyano-, or ^11^C-trifluoromethyl-labeled PET radiopharmaceuticals. The most frequently used ^11^C-labeling methods are ^*11*^*C-methylation* of amines, alcohols, thiols, carboxylates, or amides with [^11^C]CH_3_I or more reactive [^11^C]CH_3_OTf to provide *N*-, *O*-, *S*-, or in some cases *C*(sp^2^)-[^11^C]methyl-substituted products (Fig. 2a). Due to the commercial availability of modules that allow reliable production of [^11^C]CH_3_I and [^11^C]CH_3_OTf, as well as the simple labeling conditions, methylation with [^11^C]CH_3_I or [^11^C]CH_3_OTf is generally considered useful for automated synthesis not only for preclinical applications, but also in a GMP setting. A large number of PET tracers, including the clinically used [^11^C]PIB^20^ (a beta-amyloid radioligand) and [^11^C]MET^21^ (a tracer for amino acid transporters), were prepared using this method (Fig. 2a). In addition, Billard and co-workers reported the direct ^11^C-methylation of amines with [^11^C]CO_2_ under reductive conditions (ZnCl_2_, IPr, [3-bis(2,6-diisopropylphenyl)-1,3-dihydro-2*H*-imidazol-2-ylidene], and PhSiH_3_^22^. Notably, Palladium-mediated cross-coupling reactions with [^11^C]CH_3_I, including Stille coupling, Suzuki coupling, and Negishi coupling, provide access to ^11^C-methylated aromatics, such as (15*R*)-[^11^C]TIC methyl ester^23^ (a prostacyclin receptor radioligand) and [^11^C]MNQP^24^ (a serotonin transporter radioligand). Recently, MacMillan and co-workers developed the metallaphotoredox ^11^C-methylation of alkyl and aryl bromides with [^11^C]CH_3_I^25^. Both alkyl and aryl bromides can be ^11^C-methylated by photoredox/nickel dual catalysis under mild conditions.

When there is no suitable methyl group for ^11^C-labeling in the target molecule, alternative ^11^C-labeling strategies are employed, such as ^11^C-carbonylation. Carbonyl groups are commonly found in biologically active molecules, such as carboxylic acids, aldehydes, ketones, esters, amides, carbamates, and ureas. For *[*^*11*^*C]carboxylic acids* (Fig. 2b), one classic preparation method is the fixation of [^11^C]CO_2_ with strong basic Grignard or organolithium reagents. A representative example is the preparation of [carbonyl-^11^C]WAY100635^26^ (a radioligand for imaging 5-HT1A receptor), which has been used for clinical research. The high reactivity of these organometallic reagents limits the potential scope of the method, and these organometallic reagents readily absorb (^12^C)CO_2_ from the atmosphere, thereby leading to low molar activity of the tracer. Transition metal-catalyzed coupling reactions in the presence of [^11^C]CO and [^11^C]CO_2_ were efficiently employed to introduce [^11^C]carbonyl groups into various organic compounds. Indeed, Riss and co-workers reported a Cu-mediated carboxylation of boronic acid esters with [^11^C]CO_2_ in 2011^27^, whereas Vasdev, Liang and co-workers used this method in the synthesis of [^11^C]bexarotene (a retinoid X receptor agonist), with which PET/MR imaging was successfully performed in nonhuman primates^28^. Recently, Gee, Bongarzone and co-workers developed a Cu-mediated carboxylation of trialkoxysilanes and trimethylsilane derivatives with [^11^C]CO_2_ to afford [^11^C]carboxylic acids^29^. In addition, Karimi and co-workers reported on the Pd-mediated carboxylation of aryl halides/triflates and benzyl halides using [^11^C]CO in the presence of tetrabutylammonium hydroxide or trimethylphenyl-ammonium hydroxide, respectively^30^. These reactions were conducted in a micro-autoclave at 180–190 °C for 5 min using high pressure (35 MPa). Similarly, Telu and co-workers reported a novel Pd-mediated carbonylation of aryl(mesityl)iodonium salts with [^11^C]CO as an efficient approach to obtain [^11^C]arylcarboxylic acids in generally good to high yields (up to 71%)^31^. Recently, Lundgren, Rotstein, and co-workers reported the carbon isotope exchange of carboxylic acids via decarboxylation/^11^C-carboxylation enabled by organic photoredox catalysis^32^. This reaction provides a mild and rapid method for direct carboxylate exchange, but the molar activity is low. For example, [^11^C]Fenoprofen could be prepared through this method in 9.5% decay-corrected RCY (radiochemical yield^33^, the amount of activity in the product expressed as the percentage (%) of starting activity) with a molar activity of 0.029 GBq/μmol.

For [^*11*^*C*]*aldehyde and* [^*11*^*C*]*ketone* (Fig. 2c), Dahl and co-workers described a Pd-mediated ^11^C-carbonylation protocol of aryl halides or aryl triflates with [^11^C]CO using xantphos as the supporting catalyst ligand to afford [^11^C]aldehyde, [^11^C]ketone, [^11^C]carboxylic acid, [^11^C]ester, and [^11^C]amides^34^. Notably, [^11^C]formaldehyde proved to be a useful building block in the synthesis of ^11^C-labeled compounds via electrophilic aromatic substitution, Mannich-type condensations or cyclization reactions. In 2008, Hooker and co-workers reported a simple, accessible, and high-yielding (up to 89% radiochemical conversion (RCC)) method for the production of [^11^C]formaldehyde by converting [^11^C]methyl iodide to [^11^C]formaldehyde via trimethylamine *N*-oxide under mild conditions^35^. There are also other methods to access [^11^C]ketones, such as Pd-mediated ^11^C-carbonylative cross-coupling of alkyl/aryl iodides or diaryliodonium salts with organostannanes, aryl iodide or aryl triflates with boronic acids^8^.

**Fig. 3:**
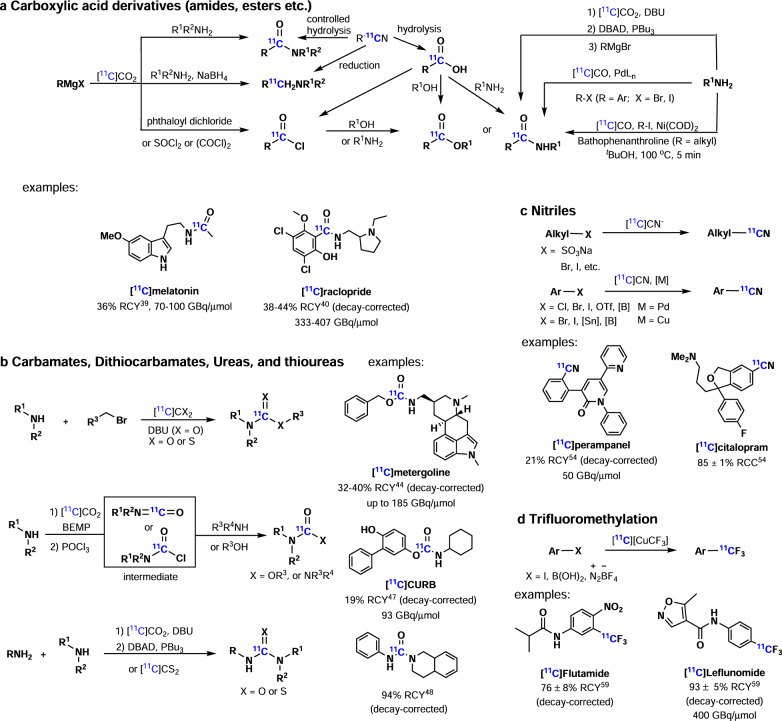
Carbon-11 Chemistry part 2. **a** Synthesis of [^11^C]carboxylic acid derivatives ([^11^C]amides, [^11^C]esters etc). **b** Synthesis of [^11^C]carbamates, [^11^C]dithiocarbamates, [^11^C]ureas, and [^11^C]thioureas. **c** Synthesis of [^11^C]nitriles. **d** ^11^C-Labeling with [^11^C]CF_3_ group.

*[*^*11*^*C]carboxylic acid derivatives* (Fig. 3a), such as [^11^C]amides, [^11^C]esters, [^11^C]amines, and [^11^C] acyl chlorides, can be easily obtained from [^11^C]carboxylic acids or [^11^C]nitriles. Among carboxylic acid derivatives, amides and esters are commonly encountered in biologically active molecules. Accordingly, several methods have been developed for their syntheses. Traditionally, [^11^C]amides could be obtained from [^11^C]carboxymagnesium halides and amine, and [^11^C]amides and [^11^C]esters could be prepared from [^11^C]acyl chlorides^36–38^. In 2017, Gee, Bongarzone and co-workers reported the rapid one-pot synthesis of [^11^C]amides from primary or activated aromatic amines with [^11^C]CO_2_ in the presence of Mitsunobu reagents via an isocyanate intermediate, followed by the addition of Grignard reagents^39^. The respective [^11^C]amides were generated in moderate RCYs, including [^11^C]melatonin (a hormone of the pineal gland), which was obtained in 36% RCY and with a molar activity of 70–100 GBq/µmol. Transition metal-mediated carbonylation cross-coupling reactions with [^11^C]CO provide an alternative pathway to afford a variety of ^11^C-labeled compounds. The main challenge is the low solubility of [^11^C]CO in organic solvents. Indeed, several strategies have been introduced to overcome this limitation, including [^11^C]CO recirculation, high-pressure reactors, continuous-flow microreactors, xenon as carrier gas (the high solubility of xenon gas in organic solvents facilitates the transfer of [^11^C]CO), [^11^C]CO-releasing molecules (such as silacarboxylic acids), solvent-soluble adducts of [^11^C]CO (such as BH_3_·[^11^C]CO), and the addition of XantPhos—a hindered bidentate phosphine ligand, found to facilitate the ^11^C-carbonylation process. Among these improved strategies, Skrydstrup, Antoni and co-workers developed pre-generated Pd-aryl oxidative addition complexes as stoichiometric reagents in carbonylation reactions with [^11^C]CO to produce structurally challenging, yet pharmaceutically relevant compounds, including [^11^C]raclopride (a dopamine D2/D3 receptor antagonist), which was obtained in 38–44% RCY (decay-corrected) and with a molar activity of 333–407 GBq/μmol^40^. The use of pre-generated Pd-aryl complexes were an excellent solution to utilize the oxidative addition process in ^11^C-carbonylation of structurally challenging substrates. The Pd-mediated ^11^C-carbonylation reactions provide an alternative access to PET tracers, but the electrophiles used in this reaction were limited to aryl, alkenyl, methyl, or benzyl halides and triflates without β-hydride to avoid the competing β-hydride elimination. In 2016, Rahman and co-workers overcame the limitation and reported the nickel-mediated ^11^C-aminocarbonylation of non-activated alkyl iodides containing β-hydrogen with [^11^C]CO at ambient pressure^41^. Ni(COD)_2_ and bathophenanthroline were used as pre-catalyst and ligand, respectively. Several aliphatic [^11^C]amides were furnished via this method in moderate to good yields. Transition metal-mediated ^11^C-carbonylation was also used to prepare [^11^C]esters (cf. recent ^11^C-carbonylation review^42^); however, the reaction scope was often limited to substrates lacking β-elimination pathway. One approach to extend the scope of accessible labeled esters entails photoinitiated free radical reactions by Långström and co-workers in the synthesis of [^11^C]carboxylic esters from a variety of primary, secondary, and tertiary alkyl iodides and [^11^C]CO^43^.

Due to their in vivo stability, *[*^*11*^*C]carbamates*, *[*^*11*^*C]dithiocarbamates*, *[*^*11*^*C]ureas*, and [^*11*^*C]thioureas* (Fig. 3b) are attractive functional groups for PET imaging ligands. In 2009, Hooker and co-workers reported a one-pot, synthesis of [^11^C]carbamates from amines and alkyl halides with [^11^C]CO_2_, employing DBU as a [^11^C]CO_2_ trapping base^44^. Several [^11^C]carbamates were synthesized via this method, including [^11^C]metergoline (antagonist of the serotonin (5HT) receptor), which was obtained in 32–40% RCY (decay-corrected) and a molar activity of up to 185 GBq/μmol. Similarly, primary amines react with [^11^C]CS_2_ very rapidly at room temperature to form the dithiocarbamate salts, followed by alkylation with alkyl halides to generate the corresponding [^11^C]thiocarbamates^45^. Of note, Miller and co-workers reported the synthesis of [^11^C]thioureas from rapid reactions between amines and [^11^C]CS_2_^46^. Wilson and co-workers reported the synthesis of [^11^C]carbamates and [^11^C]ureas with BEMP (*tert*-butylimino-2-diethylamino−1,3-dimethylperhydro-1,3,2-diazaphosphorine) as an effective [^11^C]CO_2_ trapping reagent and with POCl_3_ as the dehydrating or chlorinating reagent via the intermediate [^11^C]isocyanate^47^. A range of unsymmetrical [^11^C]carbamates and [^11^C]ureas were prepared in this one-pot, rapid procedure, including [^11^C]CURB (potent inhibitor of the enzyme fatty acid amide hydrolase), which was obtained in 19% RCY (decay-corrected) and a molar activity of 93 GBq/μmol. Similarly, the synthesis of unsymmetrical [^11^C]carbamates was also accomplished by Gee and co-workers via [^11^C]CO_2_ trapping with DBU in the presence of aliphatic and aromatic amines that reacted with Mitsunobu reagents^48^. Further, Rh-mediated ^11^C-carbonylation of azides or diazo compounds with [^11^C]CO and amines or alcohols via [^11^C]isocyanate intermediates provided another access to [^11^C]ureas or [^11^C]carbamates^49^. [^11^C]phosgene has also been used in the synthesis of [^11^C]ureas and [^11^C]carbonates (cf. recent [^11^C]phosgene review^50^). For example, a series of radiolabeled monoacylglycerol lipase inhibitors [^11^C]MAGL-0519^51^, [^11^C]SAR127303^52^, and [^11^C]TZPU^52^ were obtained from [^11^C]COCl_2_ in 6–20% RCYs with high molar activity. However, the preparation of [^11^C]COCl_2_ often requires special apparatus through the use of chlorine gas and [^11^C]CCl_4_ intermediate. Recently, Jakobsson, Pike and co-workers reported [^11^C]carbonyl difluoride ([^11^C]COF_2_) as a novel [^11^C]carbonyl group transfer agent^53^. [^11^C]COF_2_ was prepared in quantitative yields by passing [^11^C]CO gas through a AgF_2_ column at room temperature and then it was employed directly in the synthesis of a wide range of [^11^C]heterocycles, including [^11^C](*S*)-CGP-12177 (a β-adrenoceptor radioligand) and [^11^C]DMO (a radiotracer for determining tissue pH in vivo).

As nitriles are often found in biologically active molecules and are also used in the synthesis of carboxylic acids, amides, amines, and related derivatives, several ^*11*^*C-cyanation* methods have been developed (Fig. 3c). As such, aliphatic [^11^C]nitriles can be obtained by nucleophilic substitution with [^11^C]cyanide ions. The transition-metal-mediated aromatic ^11^C-cyanation reaction is an attractive approach to prepare aryl [^11^C]nitriles and has been investigated for more than 20 years. The limitation of these reactions often entails harsh reaction conditions, such as high temperatures, relatively long reaction times, and the use of inorganic bases, which limits the substrate scope. In 2015, Hooker, Buchwald and co-workers reported a rapid Pd-mediated ^11^C-cyanation of aryl halides or triflates with [^11^C]HCN and biaryl phosphine as a ligand at room temperature^54^. The sterically hindered biaryl phosphine ligands facilitated a rapid transmetalation with [^11^C]HCN and reductive elimination of aryl nitriles, whereby the reactions were completed in 1 min. A range of aryl [^11^C]nitriles was prepared via this method, including several drugs, such as [^11^C]perampanel (antiepileptic drug) obtained in 21% RCY (decay-corrected) with a molar activity of 50 GBq/μmol, and [^11^C]citalopram (antidepressant) obtained in 85% RCC. Recently, Hosoya, Zhang and co-workers developed a Pd-mediated ^11^C-cyanation of (hetero)arylboronic acids or esters with [^11^C]NH_4_CN/NH_3_ in high RCYs^55^. This method demonstrated excellent functional group tolerance and wide substrate scope. In addition, Cu-mediated ^11^C-cyanation provided an alternative access to aryl [^11^C]nitriles. Initially, Ponchant and co-workers developed the Cu-mediated ^11^C-cyanation of aryl halides, where [^11^C]cyanide ions were trapped by copper at high temperatures (180 °C)^56^. In 2017, Liang, Vasdev and co-workers developed the first Cu-mediated ^11^C-cyanation of aryl boronic acids in aqueous solutions^57^. Indeed, a broad range of arylboronic acids was labeled in 9–70% RCYs via this method. In the following year, Scott, Sanford, and co-workers further extended Cu-mediated ^11^C-cyanations to arylstannanes and other arylboron precursors with Cu(OTf)_2_^58^.

Trifluoromethyl (CF_3_) groups can be found in many drugs and potential PET radiotracers. An efficient method to incorporate ^*11*^*C-labeled trifluoromethyl group* *([*^*11*^*C]CF*_*3*_*)* with high molar activity is necessary (Fig. 3d). In 2017, Pike and co-workers reported the synthesis of [*CF*_*3*_-^11^C]trifluoromethylarenes from arylboronic acids, aryl iodides and aryldiazonium salts with [^11^C]CuCF_3_^59^. [^11^C]CuCF_3_ was prepared from [^11^C]fluoroform with copper(I) bromide and potassium *tert*-butoxide, while [^11^C]fluoroform was generated from cyclotron-produced [^11^C]methane passing over heated (270 °C) cobalt(III) fluoride (CoF_3_) in good yield. As [^11^C]methane can be generated by cyclotron with a high molar activity, a range of [*CF*_*3*_-^11^C]trifluoromethylarenes could be synthesized via this method with a high molar activity (200−500 GBq/μmol), such as flutamide (an antiandrogen drug) obtained in 76% RCY (decay-corrected) and leflunomide (an antirheumatic drug) obtained in 93% RCY (decay-corrected) with a molar activity of 400 GBq/μmol. Therefore, [*CF*_*3*_-^11^C]-trifluoromethylation with [^11^C]CuCF_3_ from [^11^C]fluoroform represents an important breakthrough for labeling PET tracers with trifluoromethyl groups with high molar activity.

#### Nitrogen-13 and Oxygen-15

Nitrogen-13 (^13^N, half-life *t*_1/2_ = 10 min) and Oxygen-15 (^15^O, half-life *t*_1/2_ = 2 min) are short-lived PET radionuclides, rendering them unsuitable for multiple-step radiosynthesis in most scenarios^8^. Of note, [^13^N]NH_3_ is the most important ^13^N-labeled tracer and it could be produced via the ^16^O(p,α)^13^N nuclear reaction^60^. Currently, [^13^N]NH_3_ has been widely used in clinical PET imaging to identify myocardial perfusion defects and assess coronary flow reserve in patients with suspected coronary artery disease. In addition, [^13^N]NH_3_ serves as a building block for further ^13^N-transformations, such as efficient enzymatic preparations of ^13^N-labeled amino acids from [^13^N]NH_3_. [^15^O]H_2_O is produced via the ^14^N(d,n)^15^O nuclear reaction^61^, and [^15^O]H_2_O is the most widely used ^15^O-labeled PET tracer and constitutes the current gold standard for cerebral blood flow measurements.

### PET chemistry of radiohalogens

#### Fluorine-18 chemistry

**Fig. 4:**
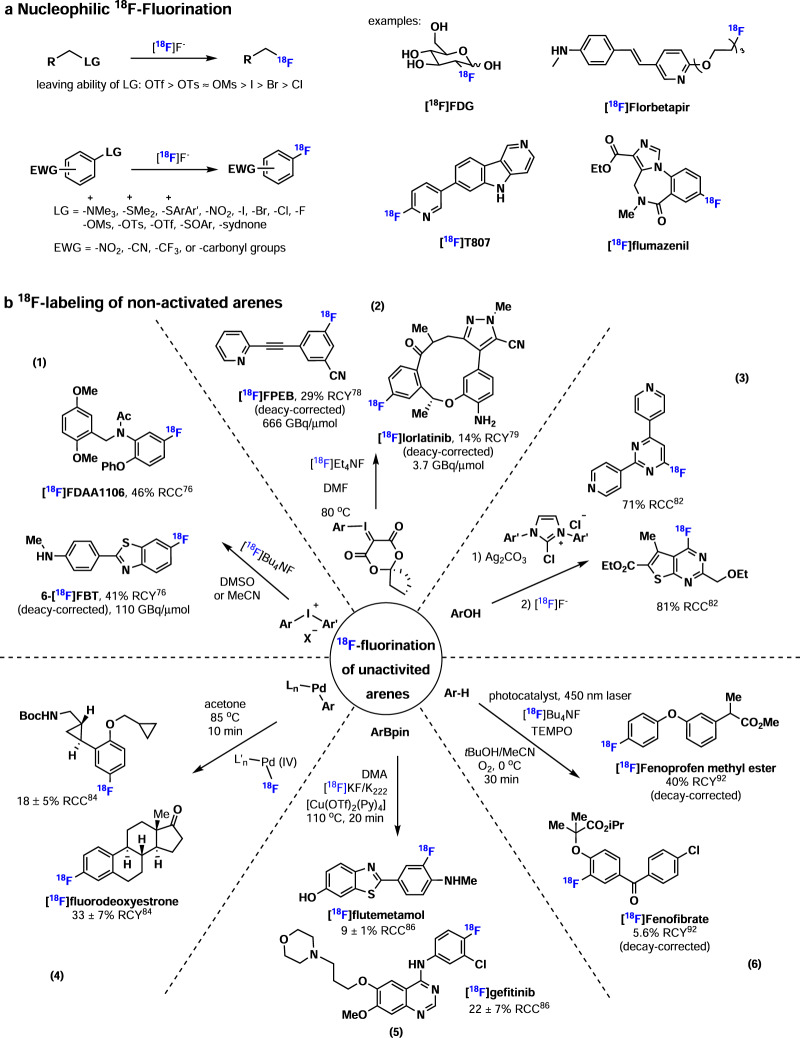
Fluorine-18 chemistry. **a** Nucleophilic ^18^F-Fluorination. **b** ^18^F-labeling of non-activated arenes.

As [^18^F]fluoride can be produced in almost all cyclotrons^62^, [^18^F]fluoride ion is the most common F-18 source to radiochemists, and most ^18^F-labeling methods start from [^18^F]fluoride. Indeed, the most frequently used method for the preparation of alkyl [^18^F]fluorides is aliphatic nucleophilic substitutions involving the displacement of a leaving group (-OTf, -OTs, -OMs, or halides etc.) with [^18^F]fluoride (Fig. 4a), where two important examples are [^18^F]FDG and [^18^F]florbetapir (a tracer for β-amyloid). Generally, nucleophilic ^18^F-fluorination is conducted in polar aprotic solvents, such as MeCN, DMF, or DMSO, to pursue high ^18^F-incorporation in a relatively short reaction time (usually ≤30 min). In a counterintuitive manner, Chi and co-workers reported an effective nucleophilic ^18^F-fluorination of aliphatic mesylates in ionic liquid and protic *tert*-butyl alcohol^63^. These reactions showed an increased ^18^F-fluorination rate and a decreased formation of undesired elimination byproducts. ^18^F-Fluorination of alcohols commonly involves two steps, including the modification of the hydroxyl group (-OH) to a leaving group (-OTs, -OMs, or -ONs) and the subsequent ^18^F-fluorination. In 2015, Doyle and co-workers reported PyFluor (2-pyridinesulfonyl fluoride) as a deoxyfluorination reagent for alcohols^64^. The ^18^F-deoxyfluorination of protected carbohydrates was achieved with [^18^F]PyFluor in 15% RCC. In addition, O’Hagan and co-workers reported the enzymatic ^18^F-fluorination of SAM [(*S*)-adenosyl-L-methionine], and the following [^18^F]-5′-FDA ([^18^F]5′-fluoro-5′-deoxy-adenosine) was obtained in an RCY of up to 95%^65^. Transition metal-mediated aliphatic ^18^F-fluorination provides mild conditions to access alkyl [^18^F]fluorides. As such, Gouverneur, and Nguyen and co-workers successfully developed Pd- and Ir-mediated allyl ^18^F-fluorination reactions^66–68^. Further, Groves, Hooker and co-workers reported the Mn-mediated ^18^F-fluorination of benzylic C−H and non-activated secondary and tertiary C−H bonds^69,70^. Doyle and co-workers developed the Co-mediated enantioselective aliphatic ^18^F-fluorination of epoxides^71^.

Generally, C(sp^2^)-^18^F bonds have higher stability than C(sp^3^)-^18^F bonds towards in vivo defluorination, and aryl [^18^F]fluorides are commonly found in a wide range of PET tracers. The most classic method to prepare aryl [^18^F]fluorides is nucleophilic aromatic substitution (S_N_Ar) with [^18^F]fluoride, involving the displacement of an appropriate leaving group (-N^+^Me_3_, - sulfonium salt, -NO_2_, -halides, etc.) with [^18^F]fluoride (Fig. 4a). Ideally, these precursors contain an electron-withdrawing functionality (-NO_2_, -CN, -CF_3_ or -C=O etc.) as an activating group in the *ortho* or *para* position to the leaving group to facilitate S_N_Ar reactions. Recently, several novel precursors for S_N_Ar reactions with [^18^F]fluoride have been developed, including triarylsulfonium salts^72,73^ and *N*-arylsydnones^74^.

A main challenge in aromatic ^18^F-fluorination is the poor reactivity of non-activated or electron-rich arenes, and efforts have been made to address this challenge (Fig. 4b). In 1995, Pike and co-workers reported the ^18^F-fluorination of diaryliodonium salts under metal-free conditions (Figs. 4b-1)^75^. In the ^18^F-fluorination of unsymmetrical diaryliodonium salts, ^18^F-fluorination preferably occurs in the relatively electron-deficient aryl group. In addition, aryl groups bearing electron-withdrawing *ortho* substituents are more amenable to radiofluorination. Several electron-rich partner aryl groups were incorporated into unsymmetrical diaryliodonium salts to achieve regioselective ^18^F-fluorination, including 2-thienyl, 4-methoxyphenyl, 3-methoxyphenyl, 4-methylphenyl and 2,4,6-trimethoxyphenyl. Electron-deficient, -neutral or -rich [^18^F]fluoroarenes were successfully prepared via this method, including [^18^F]FDAA1106^76^ (a radioligand for translocator protein), obtained in 46% RCC, and 6-[^18^F]FBT^76^ (a radioligand for β-amyloid), obtained in 41% RCY (decay-corrected) with a molar activity of 110 GBq/µmol. In 2014, Liang, Vasdev and co-workers reported the spirocyclic iodonium ylides (SCIDY) as excellent precursors in ^18^F-fluorination of non-activated arenes (Fig. 4b-2)^77^. In contrast to diaryliodonium salts, aryliodonium ylides have no counterion and therefore allow convenient purification via normal phase liquid chromatography. In addition, iodonium ylides with a spirocyclic auxiliary are crystalline solids with high stability. A wide range of aryl [^18^F]fluorides was prepared via ^18^F-fluorination of spirocyclic iodonium ylides, including the clinically validated PET tracers [^18^F]FPEB (a tracer for metabotropic glutamate receptor subtype 5), obtained in 29% RCY (decay-corrected) with a molar activity of 666 GBq/µmol^78^, and [^18^F]lorlatinib (a ROS1/ALK inhibitor), obtained in 14% RCY (decay-corrected) with a molar activity of 3.7 GBq/µmol^79^. Notably, Liang, Vasdev and co-workers further developed the second generation of iodonium ylides with a SPIAd (spiroadamantyl-1,3-dioxane-4,6-dione) auxiliary^80^. SPIAd ylide is more sterically hindered and has excellent stability under labeling conditions. A wide range of heterocycles and drug fragments were ^18^F-labeled with SPIAd ylides, including the clinically validated [^18^F]mFBG and [^18^F]FDPA, the latter of which was obtained in 23% RCY (decay-corrected) with a molar activity of 529 GBq/μmol^81^. In 2016, Ritter and co-workers reported the ^18^F-fluorination of phenols involving a concerted nucleophilic aromatic substitution (CS_N_Ar) mechanism, which was different from the classic S_N_Ar reactions via a Meisenheimer intermediate (Fig. 4b-3)^82^. This reaction was highly effective with electron-deficient phenols. Further, Ritter and co-workers extended the ^18^F-fluorination of phenols to electron-rich phenols activated by a ruthenium complex^83^.

**Fig. 5:**
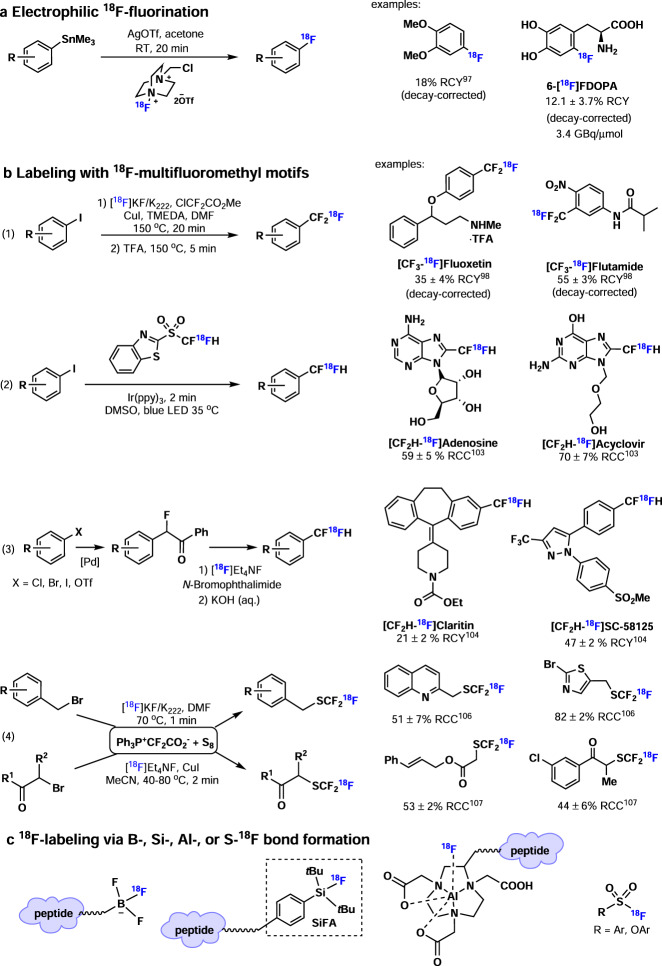
Fluorine-18 chemistry part 2. **a** Electrophilic ^18^F-fluorination. **b** Labeling with ^18^F-multifluoromethyl motifs. **c** ^18^F-labeling via B-, Si-, Al-, or S-^18^F bond formation.

**Fig. 6:**
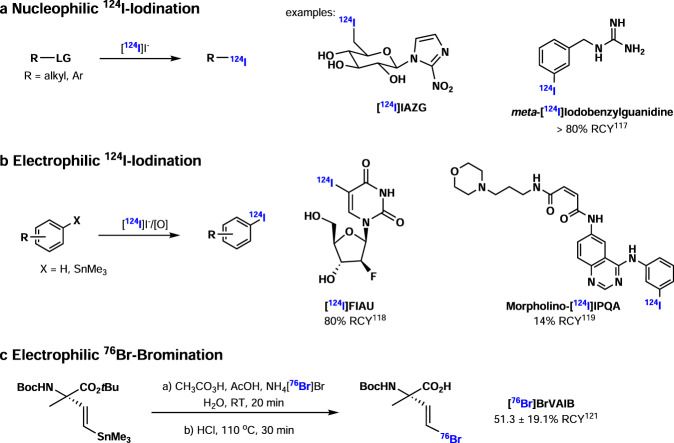
I-124/Br-76 chemistry. **a** Nucleophilic ^124^I-iodination. **b** Electrophilic ^124^I-iodination. **c** Electrophilic ^76^Br-bromination.

Transition metal-mediated ^18^F-fluorination with an enhanced activity provides a mild approach to radiofluorinate non-activated arenes. In 2011, Ritter, Hooker, and co-workers developed a Pd-mediated aromatic ^18^F-labeling method using a palladium(IV) [^18^F]fluoride complex as the electrophilic fluorination reagent (Fig. 4b-4)^84^. In particular, ^18^F-fluorination involved reductive elimination of the C−^18^F bond from a palladium(IV) aryl [^18^F]fluoride complex intermediate. Subsequently, Ritter and co-workers described a nickel-mediated oxidative radiofluorination approach with a hypervalent iodine oxidant at room temperature, requiring <1 min reaction time^85^. In 2014, Gouverneur and co-workers described a Cu-mediated ^18^F-fluorination of aryl boronic esters (aryl-BPin) (Fig. 4b-5)^86^. As aryl-BPin reagents have high stability to air and moisture, they are considered excellent precursors for ^18^F-fluorination. Indeed, the latter approach was used to radiofluorinate a large range of electron-neutral, -rich, and -poor arenes, including ^18^F-gefitinib, obtained in 22% RCC (an inhibitor of the epidermal growth factor receptor−tyrosine kinase), and [^18^F]flutemetamol (a β-amyloid-targeted probe) obtained in 9% RCC, and clinically validated [^18^F]FDOPA obtained in 10% RCY (decay-corrected) with a molar activity of 76 GBq/μmol^87^. Subsequently, Scott, Sanford and co-workers also reported Cu-mediated ^18^F-fluorination of aryl boronic esters (aryl-BPin) and aryl boronic acids^88^. Additionally, diaryliodonium salts^89^ and aryl stannanes^90^ were found to be useful precursors for Cu-mediated ^18^F-fluorination, and a range of clinically validated PET tracers was prepared via these methods, such as [^18^F]SDM-8 (a PET tracer for imaging synaptic density) was obtained from ^18^F-labeling of aryl stannane precursor in 35% RCY (decay-corrected) with a molar activity of 241.7 GBq/μmol^91^. Recently, Li, Nicewicz and co-workers developed the aryl C−H ^18^F-fluorination via organic photoredox catalysis (Fig. 4b-6)^92^. An organic acridinium-based photocatalyst was used, along with TEMPO as a redox co-mediator. Various electron-rich aromatics were ^18^F-labeled via this method, including [^18^F]fenoprofen methyl ester, obtained in 39% RCC, and [^18^F]fenofibrate (cholesterol-lowering drug), obtained in 5.6% RCY (decay-corrected). In addition, they extended this method to site-selective radiofluorination of C(sp^2^)–O bond^93^ and C(sp^2^)–halide bond^94^. A broad range of readily available aryl halide and ether precursors could be used in the synthesis of aryl [^18^F]fluoride via site-selective [^18^F]fluorination under mild photoredox conditions.

As an electrophilic ^18^F-fluorination reagent, [^18^F]F_2_ can be used as an alternative F-18 source (Fig. 5a). With non-radioactive F_2_ applied as a carrier gas, the production of [^18^F]F_2_ typically yields low molar activities (0.04 to 0.4 GBq/μmol), which limits its widespread use. To overcome this limitation, an improved method was developed, harnessing the gas phase ^19^F/^18^F isotopic exchange between [^18^F]CH_3_F and [^19^F]F_2_ using an electrochemical chamber, and yielding [^18^F]F_2_ molar activities of up to 55 GBq/μmol^95^. However, this method has proven too delicate for widespread use. Poor chemo- and regio-selectivity constitutes another main challenge in ^18^F-fluorination with [^18^F]F_2_ because of its high reactivity. Several mild electrophilic ^18^F-fluorination reagents have been synthesized from [^18^F]F_2_, including [^18^F]NFSI (*N*-fluorobenzenesulfonimide) and [^18^F]Selectfluor. Indeed, [^18^F]NFSI was synthesized and successfully employed for the enantioselective enamine-mediated ^18^F-fluorination of aldehydes by Gouverneur and co-workers^96^. Similarly, [^18^F]Selectfluor was developed by Gouverneur, Luthra, Solin and co-workers and utilized for the ^18^F-fluorination of silyl enol ether and electron-rich arylstannanes^97^—including the synthesis of 6-[^18^F]FDOPA. However, these approaches still yield tracers with low molar activities, and further work has to be conducted to extend this method for clinically relevant tracer production.

As drugs with multifluoromethyl groups, such as -CF_3_, -CF_2_H, and -SCF_3_, are increasingly being developed, there is a continuous demand for novel methods to ^18^F-label these multifluoromethyl groups (Fig. 5b). In the early days, halide/^18^F exchange reactions under harsh reactions were the main approach to ^18^F-label multifluoromethyl groups. In 2013, Gouverneur and co-workers reported a Cu-mediated ^18^F-trifluoromethylation of aryl iodides with chlorodifuoroacetate and [^18^F]fluoride, generating [^18^F]CuCF_3_ in situ (Fig. 5b-1)^98^. Various [^18^F]trifluoromethyl arenes were successfully prepared via this method, including [CF_3_-^18^F]-fluoxetine (antidepressant) and [CF_3_-^18^F]flutamide (anti-androgen). In addition, Riss and co-workers reported Cu-mediated ^18^F-trifluoromethylation using CHF_2_I to generate [^18^F]CuCF_3_^99^. Furthermore, several methods were developed to generate [^18^F]CuCF_3_ in a stepwise strategy. [^18^F]HCF_3_ was generated from [^18^F]fluoride and CHF_2_I^100^ or difluoromethyl sulfonium salt^101^ in the first step, then [^18^F]HCF_3_ was converted to [^18^F]CuCF_3,_ which was subsequently used in ^18^F-trifluoromethylation. Recently, Gouverneur and co-workers further described the radical ^18^F-trifluoromethylation of unmodified peptides at tryptophan or tyrosine residues using [^18^F]CF_3_SO_2_NH_4_ that was prepared from [^18^F]fluoride, PDFA (difluorocarbene), and SO_2_^102^. The difluoromethyl group (−CF_2_H) is also of great importance in biologically active compounds and several methods have been developed to synthesize [^18^F]difluoromethyl arenes. In 2019, Genicot, Luxen and co-workers reported the ^18^F-difluoromethylation of heteroaromatics with [^18^F]difluoromethyl benzothiazole sulfone via organic photoredox catalysis under mild conditions (Fig. 5b-2)^103^. Representative examples include [CF_2_H-^18^F]adenosine and [CF_2_H-^18^F]acyclovir, obtained in 59% RCC and 70% RCC, respectively. In addition, Ritter, Vasdev, Liang and co-workers report a practical one-pot method for the synthesis of ^18^F-difluoromethyl arenes from [^18^F]fluoride (Fig. 5b-3)^104^. At the beginning, a benzoyl auxiliary was built on aryl chloride, bromide, iodide or triflate. Then the labeling procedure entails the following steps: C-H bromination, in situ Br/^18^F exchange, and benzophenone cleavage to generate a range of highly functionalized [^18^F]difluoromethyl arenes, including [CF_2_H-^18^F]Claritin, and [CF_2_H-^18^F]SC-58125 obtained in 21% and 47% RCY, respectively. Initially, [^18^F]trifluoromethylthiol arenes were prepared via halide/^18^F exchange, whereby silver(I) was used to promote this process. Recently, Gouverneur and co-workers reported the ^18^F-labeling of unmodified peptides at the cysteine residue via [^18^F]CF_3_ group transfer using the [^18^F]Umemoto reagent^105^. In 2015, Liang, Xiao and co-workers reported the ^18^F-trifluoromethylthiolation of alkyl halides via transfer of -SC[^18^F]F_3_, generated from difluorocarbene (PDFA), sulfur, and [^18^F]fluoride under neutral conditions (Fig. 5b-4)^106^. Of note, [^18^F]CF_3_S^−^ was successfully afforded by reacting [^18^F]CF_3_^−^ with sulfur. They further extended the scope of ^18^F-trifluoromethylthiolation reactions to α-bromo carbonyl compounds (Fig. 5b-4)^107^. Intriguingly, mechanistic studies unveiled that thiocarbonyl fluoride (S=CF_2_) was generated via sulfuration of difluorocarbene with sulfur (S_8_), followed by the reaction of S=CF_2_ with [^18^F]fluoride to give [^18^F]CF_3_S^−^. The addition of Cu(I) promoted the reactions.

In addition, a number of ^18^F-labeling methods via B-, Si-, Al-, S-^18^F bond formation were developed in the past decade and reviewed recently (Fig. 5c)^8,108,109^. As B-, Si-, and Al-^18^F bonds have high bond dissociation energies, the ^18^F-labeling process is efficient in an aqueous solution, which is highly suited for labeling biomolecules for PET imaging studies. Although low pH and/or elevated temperatures are still needed for efficient ^18^F-labeling, a recent advance has been made to achieve Al-^18^F efficient labeling at physiological temperatures^110^. Compared to the C-^18^F formation via S_N_2- or S_N_Ar-type reaction, S^IV^-^18^F formation has a lower kinetic barrier, thus enabling ^18^F-labeling at room temperature as evidenced in the work of aryl [^18^F]fluorosulfates by Wu, Yang, Sharpless and co-workers, including a PARP1-targeted probe for tumor imaging^111^.

Furthermore, a stepwise and indirect ^18^F-labeling method via the link ^18^F-labeled prosthetic group to the target molecule is an attractive approach for labeling well-functionalized molecules^109^. A range of ^18^F-labeled prosthetic groups have been developed, such as 2-[^18^F]fluoroethyl tosylate^112^, and utilized in the synthesis of PET tracers, such as [^18^F]FET and [^18^F]FMeNER-*d*_2_ (deuterium atoms are incorporated to enhance the stability of the tracer). More details about the ^18^F-labeled prosthetic groups and strategies to increase the metabolic stability of radiotracers can be found in some excellent reviews^113–115^.

#### Related radiohalogens (Bromine-76 and Iodine-124 chemistry)

Iodine-124 is usually produced via ^124^Te(p,n)^124^I nuclear reaction in the cyclotron and Iodine-124 (^124^I, *t*_1/2_ = 4.2 d) has a relatively long half-life of 4.2 days, which is attractive for investigation of enduring biological processes in vivo. Although the maximum positron energy of 2.14 MeV and the positron intensity of 25% make iodine-124 not an ideal positron emitter, iodine-124 is still a clinically relevant long-lived, non-radiometal nuclide, which enables quantitative PET imaging over several days. The well-established iodine chemistry provides an excellent platform to incorporate ^124^I into organic compounds. Generally, nucleophilic ^124^I-iodination reactions can be used to synthesize ^124^I-labeled alkyl or aryl compounds via S_N_2 or S_N_Ar mechanism (Fig. 6). For example, the hypoxia imaging agent [^124^I]IAZA could be generated via a nucleophilic substitution reaction with sodium [^124^I]iodide^116^. In recent years, copper (I) salts were found to be an accelerant for halogen-^124^I exchange in reactions between [^124^I]iodide with bromo- or iodo-aryls. As an example, *meta*-[^124^I]iodobenzylguanidine (MIBG), a norepinephrine transporter (NET) imaging tracer, could be synthesized via copper-mediated nucleophilic halogen exchange reaction with sodium [^124^I]iodide in a yield over 80%^117^. Another useful method to synthesize aryl [^124^I]iodides is electrophilic ^124^I-iodination with [^124^I]iodide in oxidative conditions. [^124^I]Iodide can be easily oxidized in situ to positive iodine species which serve as an electrophile in ^124^I-iodination of aromatics or aromatic stannyl precursors. A range of ^124^I-labeled compounds was generated via this method, including [^124^I]FIAU (2′-fluoro-2′-deoxy-1-β-D-arabinofuranosyl-5-[^124^I]iodouracil, a herpes virus thymidine kinase (HSV1-tk) PET tracer) obtained in 80% RCY^118^, and morpholino-[^124^I]IPQA (EGFR kinase PET tracer) obtained in 14% RCY^119^. In addition, electrophilic ^124^I-iodination has also been used in labeling biomolecules, like peptides, antibodies, and proteins, and the presence of tyrosine, histidine, or an aromatic moiety is needed to complete such transformation. Notably, if the pH exceeds 8.5, the ^124^I-iodination of histidine is preferred^120^.

Bromine-76 is often produced via ^76^Se(p,n)^76^Br nuclear reaction in the cyclotron and bromine-76 (^76^Br, *t*_1/2_ = 16.2 h) has a medium half-life of 16.2 h, longer than fluorine-18 (*t*_1/2_ = 109.8 min) and shorter than iodine-124 (*t*_1/2_ = 4.2 d). Bromine-76 has a high positron energy (4 MeV) and its positron emission intensity is 57%. Similar to ^124^I-iodination reactions, bromine-76 could also be introduced into organic molecules via nucleophilic ^76^Br-bromination with [^76^Br]bromide or electrophilic ^76^Br-bromination with [^76^Br]bromide under oxidative conditions. In general, [^76^Br]bromide is more difficult to oxidize than [^124^I]iodide, so harsher conditions are required for oxidative ^76^Br-bromination. For example, Lapi and co-workers reported the synthesis (*S*)-amino-2-methyl-4-[^76^Br]bromo-3-(*E*)-butenoic Acid (BrVAIB) via oxidative ^76^Br-bromination of the corresponding tin precursor in the present of peracetic acid in 51% RCY, and this tracer was evaluated in PET imaging of mice with brain tumor^121^. Recently, Zhou and co-workers reported copper-mediated ^76^Br-bromination of aryl boron precursors, and a ^76^Br-labeled derivative of Olaparib (an inhibitor of PARP-1 (poly (ADP-ribose) polymerase-1)) was synthesized in 99% RCC via this method^122^.

#### Clinical examples of small molecule-based PET pharmaceuticals

**Fig. 7:**
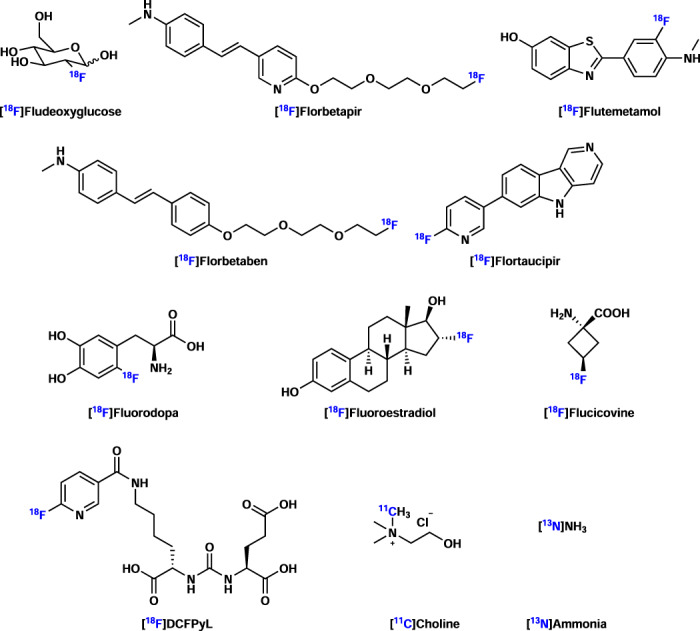
Selected FDA-approved small molecule-based PET radiopharmaceuticals. A number of small molecules labeled with F-18, C-11, and N-13 have been approved by FDA and used as PET radiopharmaceuticals.

In this chapter, we will discuss selected examples of PET radioligands that have obtained US Food and Drug Administration (FDA) approval, thereby elaborating on their clinical indications as well as their impact on patient management in clinical routine (Fig. 7). While PET radiopharmaceuticals are of paramount value in both diagnostic imaging and drug discovery^123^, the majority of contemporary FDA-approved small molecule PET radiopharmaceuticals are labeled with fluorine-18, which is attributed to its optimal decay properties, including a relatively clean positron decay, a short positron range as well as a physical half-life that allows satellite distribution^5^. Since the first human PET studies in the 1970s, [^18^F]FDG has been the cornerstone of PET imaging. Indeed, as an imaging biomarker for glucose metabolism, [^18^F]FDG has been widely used for patient diagnosis, disease staging, and therapy monitoring of various pathologies in oncology, neurology, and cardiology.

Although [^18^F]FDG remains the most widely used PET tracer in the clinic, there is a continuous demand for more specific probes that are highly selective for a given biological target. As such, over the past several decades, PET has provided unique and powerful insights into normal brain function as well as various neurological disorders and neurodegenerative diseases via probes that accumulate at the sites of misfolded protein aggregates. For instance, Alzheimer’s disease (AD) constitutes a progressive neurodegenerative disease that is typically characterized by extracellular amyloid-beta (Aβ) plaques and intracellular neurofibrillary tangles that are formed by misfolded tau aggregates^124^. Notably, the ground-breaking discovery of Aβ-targeted PET ligand, [^11^C]PIB, and its successful application for the early diagnosis of AD fueled the development of radiofluorinated analogs, [^18^F]florbetapir, [^18^F]flutemetamol, and [^18^F]florbetaben, all of which obtained FDA approval for the diagnosis of AD between 2012 and 2014. It should be noted that Aβ-targeted PET has substantially improved the diagnostic accuracy (the presence or absence of Aβ in the living brain) of AD in clinical routine, exhibiting a remarkably high predictive value. Similarly, a series of tau-targeted PET tracers have been developed. Among them, [^18^F]flortaucipir was the first PET tracer approved by the FDA for imaging aggregated tau neurofibrillary tangles in 2020^125^. It is anticipated that tau pathology can be detected early in the disease course, thus enabling more clinical findings by tau-targeted PET compared to Aβ-targeted PET in the course of AD progression. Parkinson’s disease (PD) is a long-term degenerative disease that mainly affects the motor system. [^18^F]Fluorodopa ([^18^F]FDOPA) was approved by FDA for imaging dopaminergic nerve terminals in the striatum of patients with suspected Parkinsonian syndrome in 2019^126^.

In cardiology, PET imaging is a powerful tool for in vivo evaluation of myocardial function. Among other PET probes, [^13^N]NH_3_ was approved by the FDA for rest/stress myocardial perfusion imaging (MPI) to detect ischemic heart disease. The strength of PET-MPI lies in the ability to measure absolute myocardial blood flow at rest and following pharmacologically induced stress, which allows the assessment of perfusion defects in larger coronary arteries, as well as the calculation of coronary flow reserve—an indicator of microvascular function. Notably, PET has been established as the clinical reference standard for the quantification of myocardial perfusion^127^.

In oncology, non-invasive tumor imaging provides crucial advantages in the assessment of tumor progression and regression. In 2020, [^18^F]fluoroestradiol was approved by the FDA for imaging estrogen receptor (ER)-positive breast cancer lesions^128^. Prostate cancer is common cancer among men, and PET imaging is a powerful tool for monitoring the progression or recurrence of prostate cancer^129^. Indeed, [^11^C]choline was the first FDA-approved PET tracer that was employed for the detection of recurrent prostate cancer. Similarly, [^18^F]fluciclovine, an ^18^F-labeled analog of L-leucine, was approved by the FDA for imaging prostate cancer recurrence. Although these probes proved to be useful in prostate cancer patients, their accumulation reflected the increased amino acid turnover of tumor cells; however, their mechanism of uptake was not related to tumor-specific markers. In contrast, the more recently FDA-approved prostate-specific membrane antigen (PSMA)-targeted PET radioligand, [^18^F]DCFPyL (Pylarify®), proved to be highly selective for PSMA-positive tumor cells and as such, provide high accuracy for the detection of PSMA-positive lesions in men with prostate cancer.

### PET chemistry of radiometals

#### PET Radiometals

**Fig. 8:**
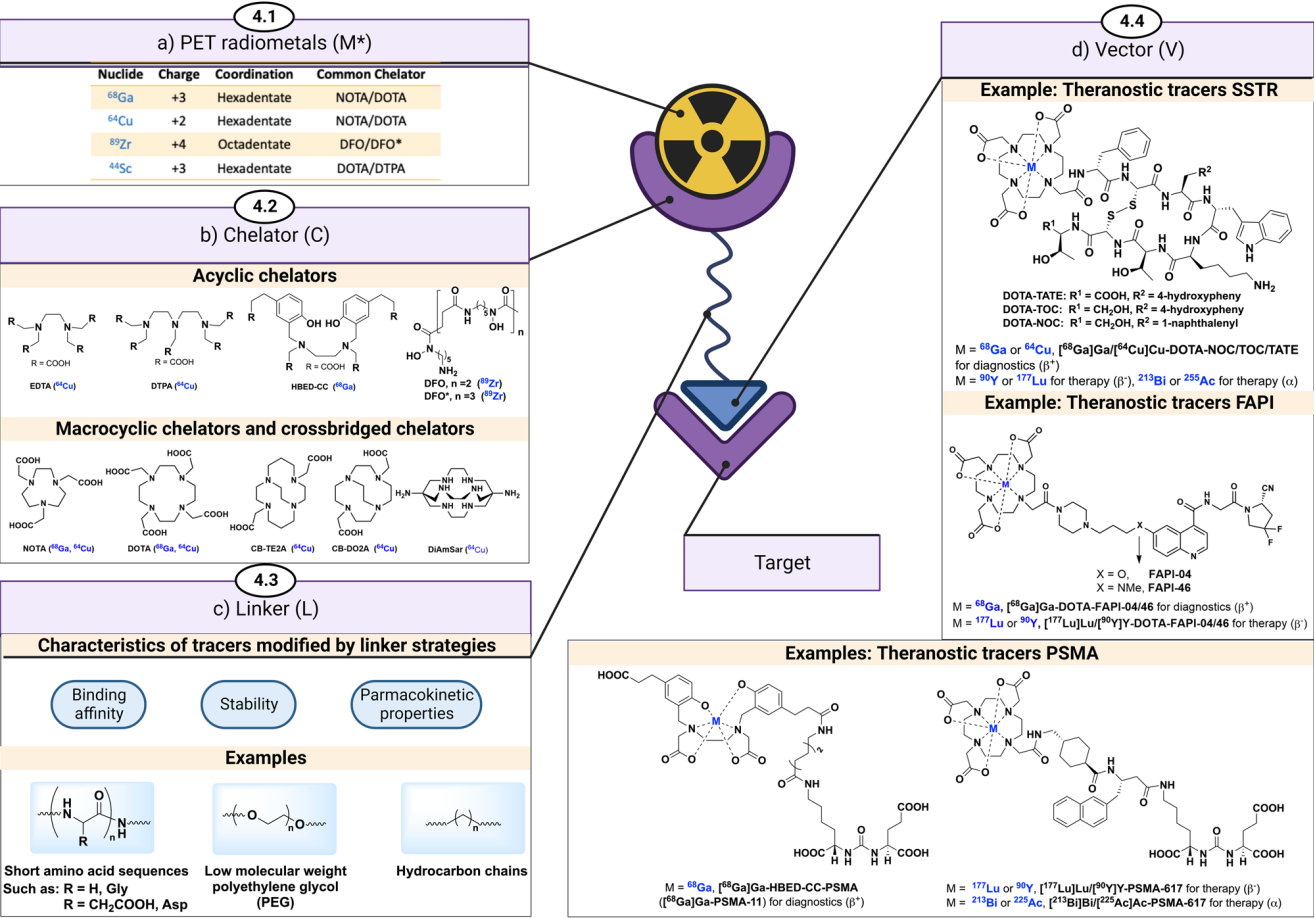
Radiometal chemistry for PET. **a** Schematic diagram for radiometal-based PET radiopharmaceuticals (M*-C-L-TV). **b** Chelators in radiometal-based PET pharmaceuticals (acyclic chelators, cyclic chelators, and cross-bridged cyclic chelators). **c** Likers in metal-based radiopharmaceuticals (short sequences of amino acids, polyethylene glycols (PEG), and hydrocarbon chains). **d** Clinical examples of radiometal-based PET pharmaceuticals. Created with BioRender.com.

Besides organic PET radionuclides, radiometals provide new possibilities to radiolabel targeted vectors for PET which typically consists of biological molecules (e.g., peptides, proteins, and antibodies). Metal-based radiopharmaceuticals (Fig. 8a) often include a radiometal (M*) bound to a chelator (C), which is attached to a targeting vector (V) through a linker (L) in the format of M*-C-L-V. Compared to other PET radionuclides, radiometals generally have a straightforward labeling process that relies on coordination chemistry. Further, the relatively mild labeling conditions make radiometals suitable for labeling complex biological molecules. Notably, PET radiometals have a wide range of physical half-lives, ranging from hours to several days, such as gallium-68 (^68^Ga, *t*_1/2_ = 67.7 min), copper-64 (^64^Cu, *t*_1/2_ = 12.7 h), and zirconium-89 (^89^Zr, *t*_1/2_ = 78.4 h), which provides a broad radionuclide scope with suitable physical half-life to match the intended application. As such, short-lived radiometals can be used for vectors with fast pharmacokinetics and long-lived radiometals to visualize long-lasting biological processes.

An increasingly utilized feature of PET imaging is radiotheranostics. When a radiometal-based tracer is used in PET imaging for diagnosis, the following radiotherapy could be conducted by changing M* from a positron emitter (^44^Sc, ^64^Cu, ^68^Ga, or ^89^Zr) to an alpha emitter (^225^Ac, ^212^Pb, or ^213^Bi)^130,131^ or beta emitter (^177^Lu, ^90^Y, or ^67^Cu) for radiotherapy^132^. Theranostic pairs are also feasible without radiometals, for example, with Iodine isotopes^133^, or between [^18^F]MFBG^134^ and [^211^At]MABG^135^, but the inherent potential in the theranostic approach with radiometals and chelators is more modular. Of note, contemporary concepts and potential future developments in radiotheranostics have been recently reviewed by Weissleder and co-workers^133^. We will focus on the commonly used radiometals in PET, including ^68^Ga, ^64^Cu, and ^89^Zr. Other radiometals are discussed elsewhere^136,137^.

^68^Ga (*t*_1/2_ = 67.7 min) can be produced by commercially available generators (^68^Ge/^68^Ga) and is easily accessible to radiochemists. Owing to the long half-life of the parent isotope ^68^Ge (*t*_1/2_ = 271 days), ^68^Ge/^68^Ga generators can typically be used within one year. The different chemical properties between ^68^Ga and ^68^Ge make the separation possible. In the past ten years, several excellent ^68^Ga-based radiopharmaceuticals have been developed for clinical use. The accessibility and potential of these radiopharmaceuticals make ^68^Ga attractive and promising in PET. It should be noted, however, that the limited capacity and supply of GMP-grade ^68^Ge/^68^Ga generators has prompted the search for improved ways to generate gallium-68 via cyclotron. As an important improvement and supplement to the generator method (usually at ≤100 mCi capacity), the cyclotron-based method could produce ^68^Ga in high quantity up to multi Ci levels, which provides strong support for the successful use of ^68^Ga in the clinic, especially for a larger patient population^138^. ^64^Cu can be produced by different methods via a reactor, an accelerator or a cyclotron. Highly enriched ^64^Ni is used in the ^64^Ni(p,n)^64^Cu nuclear reaction within a cyclotron to produce carrier-free ^64^Cu in high purity, yield, and molar activity^139^. Unlike other PET radiometals, [^64^Cu]Cu^2+^ is not redox-inert under physiological conditions. Indeed, [^64^Cu]Cu^2+^ can be reduced to [^64^Cu]Cu^+^ under reducing conditions in vivo, which represents a practical consideration during PET tracer design and/or chelator selection (vide infra). In certain cases, the redox dynamics of copper offer some advantages. For example, this redox behavior is the mode of action for [^64^Cu]Cu-ATSM^140^, a hypoxia imaging tracer. In hypoxic regions, Cu(II) is reduced and subsequently trapped, which can be visualized via PET. Of note, the smaller positron range of ^64^Cu compared to ^68^Ga renders tracers based on ^64^Cu applicable for pathologies with higher spatial requirements, such as cardiovascular diseases.

^89^Zr is typically produced via the ^89^Y(p,n)^89^Zr nuclear reaction in a cyclotron. With a relatively long half-life (*t*_1/2_ = 78.4 h), ^89^Zr can be transported to nuclear medicine facilities without an on-site cyclotron and can be used in antibody-based PET imaging (immuno-PET). Recently, ^44^Sc has gained attention as a promising PET radionuclide due to its clean β^+^-decay (94%) and suitable physical half-life (*t*_1/2_ = 4 h). ^44^Sc can be produced either via ^44^Ca(p,n)^44^Sc in a cyclotron or by ^44^Ti/^44^Sc generators.

#### Chelators

Contrary to the labeling with other PET radionuclides involving covalent bond formation (e.g., ^18^F-C bond), labeling with radiometals requires coordination with chelators (Fig. 8b). Even if certain chelators prefer some radiometals over others, no chelator is fully metal-specific and will bind other non-radioactive metals, if present during radiolabelling. Chelators are essential to ensure (1) fast complexation of the radiometal at low temperatures, low substrate concentrations, and physiological pH, and (2) thermodynamic and kinetic stabilization of radiometals by forming inert complexes. In general, chelators can be classified in three types, acyclic chelators, cyclic chelators, and cross-bridged chelators.

*Acyclic chelators* provide fast complexation kinetics at room temperature and physiological pH. Nonetheless, acyclic chelator-metal complexes, in general, exhibit less favorable stability than the cyclic ones. The earliest chelators for radiometals were acyclic, such as the polycarboxylate chelators EDTA and DTPA. Although these chelators exhibited fast complexation kinetics at room temperature, the complexes were plagued by their low stability in vivo. Further, EDTA and DTPA have low selectivity for cations with a similar valence; however, they bind M^3+^ with a higher affinity than M^2+^. The acyclic chelator HBED-CC is unique in its use of two lipophilic phenolic groups for [^68^Ga]Ga^3+^ coordination^141^. The [^68^Ga]Ga-HBED-CC complex consists of two negatively charged carboxyl groups, two neutral nitrogen atoms and two negatively charged phenolic oxygens, creating a complex with one negative charge and with a more hydrophobic character (from the benzyl rings of the phenolic groups), as compared to other acyclic chelators. The acyclic chelator desferoxamine B (DFO)^142^ has been developed and recognized as a gold standard chelator for Zr^4+^ in the past decade. DFO is an iron-binding siderophore from the bacteria *Streptomyces pilosus*, which was approved by FDA for iron-overload disease, using the six oxygens of three hydroxamate groups to bind Zr^4+^ in a [^89^Zr][Zr(DFO)(H_2_O)_2_]^+^ complex. Escorcia and co-workers recently showed that [^89^Zr]Zr-DFO-onartuzumab, a monoclonal antibody targeting the Met receptor tyrosine kinase, can be used to predict the therapeutic response to targeted radioligand therapy in a rodent model of pancreatic cancer that was resistant to Met kinase inhibitors^143^. With the long biological and physical half-lives of [^89^Zr]Zr-DFO-mAbs (mAb, monoclonal antibody), the chelation stability of [^89^Zr]Zr-DFO-mAb needs improvement. Hence, there is an ongoing effort to obtain ^89^Zr chelates with improved stability. Notably, Mindt, Gasser, and co-workers developed a novel chelator DFO* by adding one more hydroxamate donor to DFO, and the resulting two more coordination sites of DFO* largely improved the stability of [^89^Zr]Zr-DFO* complexes^144^.

To improve the stability of M*-C complexes, *cyclic chelators* have been designed to strengthen the binding of radiometals. Cyclic chelators have more rigid chemical structures, in which the donor atoms are in a more optimal position to chelate certain radiometals. Cyclic complexes are generally more stable than acyclic complexes. Although many chelators react with radiometals at low temperatures, albeit at lower radiochemical yields, higher temperatures (40–100 °C) are typically needed for rapid radiometal binding. A few exceptions can also be found in the cases of [^64^Cu]Cu-NOTA, [^64^Cu]Cu-DiAmSar, and [^68^Ga]Ga-PCTA^136,137^. The workhorse cyclic chelator is DOTA, with many commercially available DOTA derivatives that can be easily conjugated to peptides on the solid phase. A large number of trivalent radiometals bind sufficiently tight to DOTA, including [^68^Ga]Ga^3+^ and [^44^Sc]Sc^3+^ as positron emitters for PET, [^177^Lu]Lu^3+^ and [^90^Y]Y^3+^ as beta emitters for therapy, as well as [^225^Ac]Ac^3+^ and [^213^Bi]Bi^3+^ as alpha emitters for therapy. Thus, DOTA is an excellent chelator for radiopharmaceuticals and/or the chelator of choice during the design because it allows the use of trivalent imaging radiometals (e.g., [^68^Ga]Ga^3+^), followed by treatment with therapeutic radiometals (e.g., [^177^Lu]Lu^3+^, [^225^Ac]Ac^3+^). With a smaller ring structure, NOTA is a more stable chelator than DOTA with [^68^Ga]Ga^3+^ and [^64^Cu]Cu^2+^. Further, NOTA (with one pendant arm in use for coupling to the linker/vector) is still the chelator of choice for binding Al[^18^F]F, despite elevated temperature (90–110 °C) being needed, allowing chelator-mediated ^18^F-labeling of proteins and peptides without formation of a covalent ^18^F-C bond. It is worthy of note that recently, related to Al[^18^F]F labeling, novel scaffolds HBED-CC and RESCA1, as acyclic chelators, have shown promise for Al[^18^F]F chelation at room temperature^110,145^, which is advantageous for biologics that are sensitive to elevated temperatures (>37 °C).

*Cross-bridged cyclic chelators* (Fig. 8b) have been developed to provide additional rigidity over their cyclic counterparts of similar ring dimensions, in most cases cross-bridging between N atoms and reducing the number of carboxyl groups available for radiometal binding. In general, the complexation process requires higher temperatures than cyclic chelators. [^64^Cu]Cu-CB-DO2A and [^64^Cu]Cu-CB-TE2A were found to have superior in vivo stabilities to DOTA and TETA. Cross-bridged chelators are widely used with [^64^Cu]Cu^2+^, possibly owing to the aforementioned redox potential of [^64^Cu]Cu^2+^, requiring more stable metal-chelate complexes to ensure continued binding, even on reduction. In addition, DiAmSar is a sarcophagine type chelator that can be labeled with [^64^Cu]Cu^2+^ at room temperature. The DiAmSar chelator shows an even higher in vivo stability than most other cross-bridge cyclic chelators^146^. Another sarcophagine analog, MeCOSAR^147^, was used in the synthesis of [^64^Cu]Cu-SARTATE in the clinical trial (NCT04438304) for imaging patients with known or suspected neuroendocrine tumors. Other chelators for radiometal labeling include, but are not limited to, DOTA variants, NOTA variants, TE2A variants, PCTA, macropa, HOPO, sulfur-containing chelators (e.g., ECD), DATA analogs, DTPA analogs (e.g., CHX-A”-DTPA), octapa and etc. A comprehensive discussion of these chelators is beyond the scope of the present review and can be found elsewhere^136,146,148^.

#### Linkers

Linkers are important for radiometal-based radiopharmaceuticals, as a direct coupling of the chelator and vector may result in the reduction of receptor affinity. To address this problem, a linker can be introduced between the chelator and the vector. Further, the linker can be utilized to fine-tune the physical properties of the tracer to affect in vivo behavior. An ideal linker does not negatively affect the vector affinity towards the target (sometimes even increases the binding affinity) but can be used to improve the pharmacokinetic properties of the tracer. While linkers have not been studied to a greater extent for radiopharmaceuticals, and the reports are not conclusive, the lessons we learned from antibody-drug-conjugates demonstrated that the appropriate linker depends highly on vector and chelator^149^.

Properties of linkers are critical because a suitable linker may improve radiotracer pharmacokinetics properties. There are several commonly used linkers, such as short sequences of amino acids, polyethylene glycols (PEG), and hydrocarbon chains (Fig. 8c). Simple hydrocarbon chains tend to increase the lipophilicity of radiotracer with an unfavorably slow clearance via the hepatobiliary pathway to follow. In contrast, linkers of amino acid sequences tend to increase the hydrophilicity of radiotracer with primary excretion via the renal-urinary pathway as a consequence. Generally, a radiotracer needs to achieve a high target accumulation and a high target-to-background ratio in the shortest possible time. This means that the radiotracer needs to reach the target and be cleared from non-target tissues quickly, while still matching the binding kinetics of the vector. The introduction of PEG linkers to the radiotracers was found to increase receptor-mediated uptake and renal-urinary clearance, decrease non-specific binding, and achieve a high target-to-background ratio, in most cases. As an example, the introduction of PEG linker in [^64^Cu]Cu-DOTA-PEGn-AVP04-50 could significantly decrease kidney uptake, and increase tumor uptake, compared to the non-PEGylated counterpart^150^. And for [^64^Cu]Cu-NOTA-PEGn-trastuzumab^151^, the clearance of PEGylated-radiotracer was three-fold faster while maintaining comparable tumor uptake compared to its non-PEG counterpart. In addition, some functional moieties, including albumin binders, can be introduced to the linker to improve the drug pharmacokinetics. For example, Evans Blue has been introduced to the linker to help the drugs’ binding to albumin, extend the half-life, and decrease renal clearance^152^. In all, the interplay of vector, linker, and radiometal-chelator determines in vivo imaging properties of the tracer. While the radiometal, chelator and vector are often pre-determined, the linker is easier to modify at late stage, making it an ideal component for fine-tuning and achieving optimal physicochemical properties of radiometal-based pharmaceuticals.

#### Clinical examples of radiometal-based PET pharmaceuticals

Although ^99m^Tc-labeled radiopharmaceuticals have historically dominated the landscape of radiometal-based clinical applications, the enormous potential of radiometals in nuclear medicine goes well beyond these conventional probes. Indeed, the clinical use of gallium-68 has experienced remarkable growth with recent examples of successful phase III clinical trials—particularly in oncology^133,153^. The representative examples include somatostatin receptors (SSTRs)-targeted probes in neuroendocrine tumors and PSMA-targeted probes in prostate cancer (Fig. 8d). Even though there are many reports on antibodies labeled with ^89^Zr or ^64^Cu, some of which are currently in clinical trials, none of these has been FDA approved yet.

SSTRs are receptors for somatostatin, a small neuropeptide associated with neural signaling. Overexpression of SSTRs on the cell membrane is a unique feature of neuroendocrine tumors, which is a promising target for imaging diagnosis and radiotherapy of neuroendocrine tumors. In the past decade, a range of somatostatin analogs have been developed for SSTRs PET imaging and targeted radiotherapy of neuroendocrine tumors, and the commonly used tracers include [^68^Ga]Ga-DOTA-TATE, [^68^Ga]Ga-DOTA-TOC, and [^68^Ga]Ga-DOTA-NOC^154^. In addition, DOTA-TATE/TOC/NOC was further used in targeted radiotherapy of neuroendocrine tumors after labeling with alpha emitter (^213^Bi) and beta emitter (^177^Lu or ^90^Y). Notably, [^68^Ga]Ga-DOTA-TATE (NETSPOT^TM^), [^177^Lu]Lu-DOTA-TATE (Lutathera®), and [^68^Ga]Ga-DOTA-TOC were approved by the FDA in 2016, 2018, and 2019, respectively^155^.

PSMA has a very low expression in normal prostate cells, but a high expression in prostate cancer cells, and the expression level is directly proportional to prostate cancer progression. Hence, PSMA is a promising target for imaging diagnostics and targeted radionuclide therapy for prostate cancer and its metastases. Currently, various PET tracers have been developed for the diagnosis and treatment of prostate cancer, such as [^68^Ga]Ga-PSMA-HBED-CC (also known as [^68^Ga]Ga-PSMA-11)^156,157^, and [^18^F]PMSA-1007^158^. In addition to [^18^F]DCFPyL (Pylarify®; cf. section 3.3, FDA approved ^18^F radiopharmaceuticals), [^68^Ga]Ga-PSMA-11 is another widely used radiotracer in clinical research and has been approved by the FDA in December 2020 for the imaging of PSMA-positive lesions in men with prostate cancer^159^. [^68^Ga]Ga-PSMA-11 exhibited high stability and a high uptake in prostate cancer cells. Another ligand PSMA-617 was developed for radionuclide therapy with ^177^Lu (*t*_1/2_ = 6.7 d). Indeed, [^177^Lu]PSMA-617 was granted FDA breakthrough designation in castration-resistant prostate cancer after both primary clinical endpoints of overall survival and radiographic progression-free survival were met in the VISION trial (NCT03511664) and received FDA approval in March 2022^160,161^.

Fibroblast activation protein (FAP) is highly expressed in more than 90% of epithelial tumors, which makes FAP a promising target for diagnosis and therapy of various types of cancers. Notably, the FAP-targeted probes, [^68^Ga]Ga-DOTA-FAPI-04 and [^68^Ga]Ga-DOTA-FAPI-46, were introduced as suitable radioligands for imaging different cancers and demonstrated high tumor uptake and fast clearance from the normal tissue, resulting high image quality^162,163^. Further, the universal DOTA chelator, in principle, warrants the use of DOTA-FAPI-04 and DOTA-FAPI-46 for theranostics. Although [^68^Ga]Ga-DOTA-FAPI-04 and [^68^Ga]Ga-DOTA-FAPI-46 have not yet been approved by the FDA, the results obtained so far have been promising.

More recent examples of radiometal-based PET pharmaceuticals that were advanced to human research include the angiotensin-converting enzyme 2 (ACE2)-targeted probes, [^64^Cu]HZ20, and [^68^Ga]HZ20^164^. Notably, ACE2 constitutes a key viral gateway that facilitates SARS-CoV-2 entry into host cells^165–167^. As such, it was hypothesized that non-invasive quantification on ACE2 abundancy in the lungs and other organs holds promise to deliver prognostic value and support the development of ACE2-targeted therapeutic intervention^164^. [^64^Cu]HZ20 and [^68^Ga]HZ20 exhibited nanomolar potency and showed favorable pharmacokinetics in a fist-in-human study with 20 healthy volunteers. Future studies with [^64^Cu]HZ20 and [^68^Ga]HZ20 will shed light on the diagnostic and prognostic value of ACE2-targeted PET in COVID-19 patients. Recently, [^89^Zr]Zr-Atezolizumab has been used as a promising PET tracer for assessing clinical response to PD-L1 blockade in cancers^168^. PET imaging with [^89^Zr]Zr-Atezolizumab before and after the therapy might be of great usefulness for determining PDL-1 expression in tumors.

### Bioconjugation in PET tracer development

**Fig. 9:**
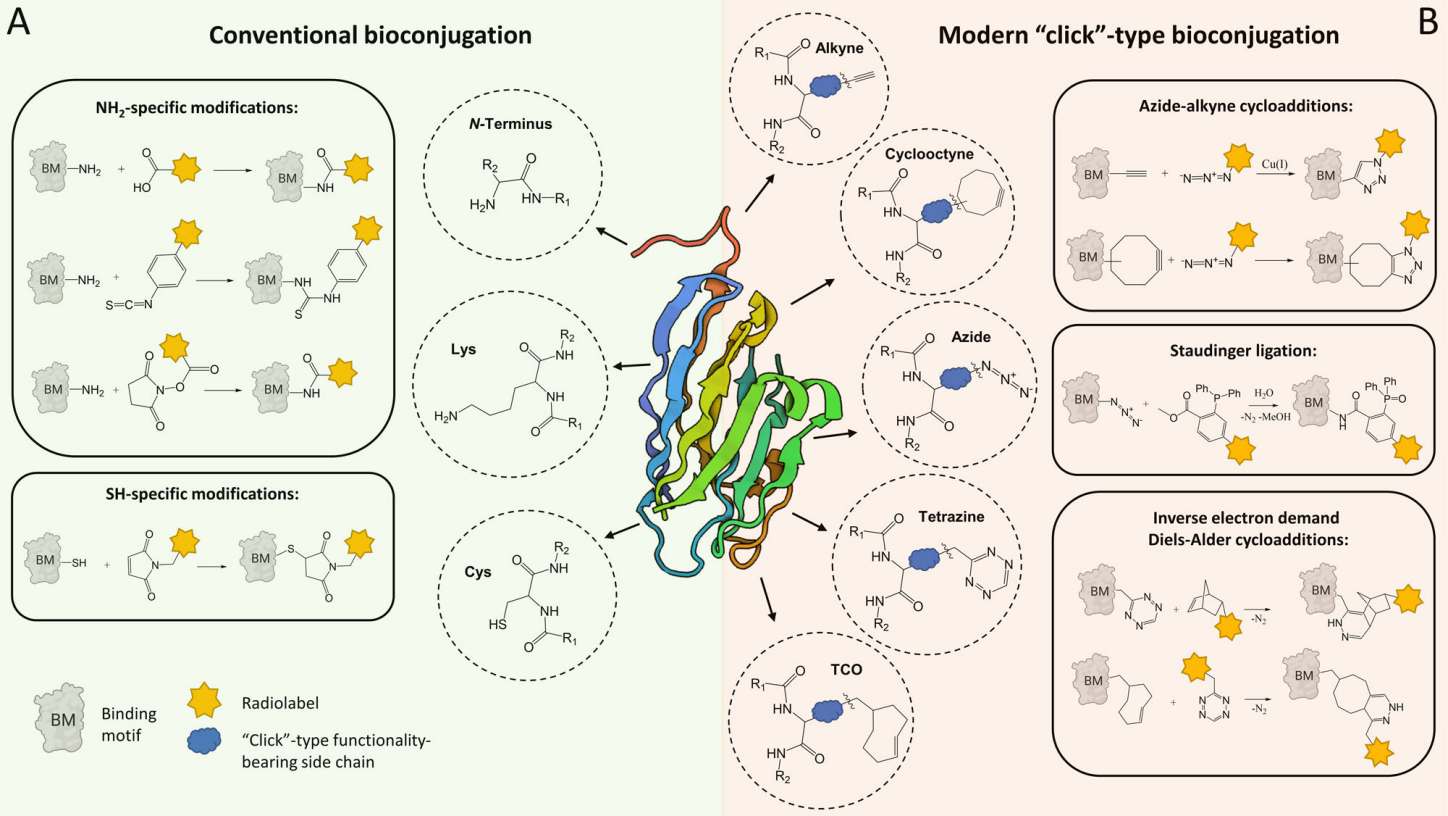
Conventional vs. modern (Click-type) bioconjugation chemistry. **A** While peptides and proteins were traditionally labeled via NH_2_- and thiol-specific modification with *N-*hydroxysuccinimides (NHS), isothiocyanates, or maleimides, respectively, **B** modern (Click-type) bioconjugation strategies involve fast and high-yielding orthogonal radiochemistry with non-naturally occurring chemical functionalities. Created with BioRender.com.

Herein, we refer to bioconjugation as a general chemical process to form a covalent bond between two molecules, of which at least one is a biomolecule (can be found in biological systems). Of note, biomolecules such as antibodies, antibody fragments and peptides have become a crucial component of modern drug development. The continuously growing number of FDA approvals for therapeutic biomolecules has prompted enthusiasm to further exploit the use of biomolecules for diagnostic purposes^169,170^. While PET radiometals have traditionally been used in combination with antibodies, novel radiochemical tools have widened the radiolabeling scope, particularly with regard to non-metal radionuclides^171,172^. Bioconjugation reactions can be subdivided into conventional methods, that use primary amine with *N-*hydroxysuccinimides (NHS), isothiocyanates and anhydrides as well as thiol modifications with maleimides, and modern click-type bioconjugation methods, which are typically based on orthogonal reactions including, but not limited to, azide-alkyne cycloadditions, Staudinger ligations and inverse electron demand Diels-Alder cycloadditions (Fig. 9)^173^. Orthogonal reactions are highly selective between a pair of chemical functionalities and can proceed with high yields in aqueous media and at ambient temperature^174,175^. The employed chemical functionalities are typically not present in nature, which allows specific reactions in the presence of naturally occurring functional groups. When orthogonal reactions are inert to biological moieties, they are termed bioorthogonal. Indeed, bioorthogonal reactions constitute the cornerstone of pretargeted imaging, where two components, namely the functionalized targeting vector and the radioisotope-bearing prosthetic group, are administered separately and react in vivo^176^. In this chapter, we will discuss conventional and modern click-type bioconjugation methods, thereby highlighting recent advances in orthogonal PET chemistry for fast and efficient in vitro/in vivo applications.

#### Conventional bioconjugation

Conventional bioconjugation methods harness the reactivity of amines and thiols, which are present in natural amino acids such as lysine and cysteine residues as well as the *N-*terminal residues (Fig. 9a). Traditionally, the radiolabeling of proteins was accomplished by reacting their amine or thiol functionalities to chelator-bearing reactive electrophiles and subsequent chelation of the radionuclide, or reaction with radiolabelled prosthetic groups^148^. Indeed, the vast majority of traditional bioconjugation examples involve the reaction of cysteine residues with radiolabeled maleimides via Michael addition or the modification of primary amines with radiolabeled NHS esters, isothiocyanates and activated carboxylic acid derivatives^177–181^. Furthermore, these reactions are mainly not site-specific since biomolecules such as antibodies contain multiple primary amines (lysines), resulting in the difficulty of controlling bioconjugate sites, potentially impacting their binding properties. Despite their widespread use, conventional bioconjugation reactions offer room for improvement, particularly with regard to reaction kinetics, radiochemical yields and stability issue with maleimide adducts (via retro-Michael addition)^173^.

#### Modern click-type bioconjugation

In the past, the vast majority of bioconjugation concepts have relied on conventional bioconjugation reactions. To overcome the previously mentioned limitation of conventional bioconjugation methods, however, radiochemists have increasingly focused on labeling strategies that involve orthogonal clickable prosthetic groups (Fig. 9b). Indeed, a variety of click-type bioconjugation methods have been developed, and in some cases, provided more optimal reaction kinetics, higher radiochemical yields, or more stable labeled vectors. Orthogonal reactions that rely on naturally occurring primary amines do not provide site-selective radiolabeling since biomolecules typically bear multiple primary amines. Nonetheless, the high chemical selectivity of orthogonal reactions provides a more uniform tracer, thereby easing a subsequent regulatory process^173^. Among the orthogonal reactions harnessed by radiochemists, azide-alkyne cycloadditions (AACs) have been most extensively employed for peptide and protein labeling^182–194^. A unique advantage of this reaction is that the click product, a 1,2,3-triazole ring, is well tolerated by the binding domain due to its relatively small and rigid structure, which essentially forms the bioisostere of an amide linkage. In light of the highly suited AAC properties for fast and efficient radiolabeling, a wide variety of radiofluorinated alkyne- or azide-bearing prosthetic groups were harnessed with great success, yielding numerous radiolabeled vectors including peptides, oligonucleotides, and proteins^195–205^.

Notwithstanding the successful implementation of CuAAC in PET radiochemistry, this reaction has some shortcomings, namely, the need for a copper catalyst and the presence of a reductant (commonly ascorbic acid), both of which may interact or react with the vector as well as the radiolabeled prosthetic group. Particularly, serine, histidine, and arginine residues can coordinate copper cations, imposing potential structural and functional changes on the vector^198,205–207^. Further, residual copper(II) ions may displace the far less abundant PET radiometals from their chelators^208^. In sharp contrast, second-generation AACs are catalyst-free and, hence, better tolerated by biomolecules^173^. Indeed, by employing ring strain-activated cyclic alkynes, such as dibenzocyclooctyne, the reaction can be accomplished in aqueous solutions, at ambient temperature and without copper catalyst^209,210^. This strain-promoted azide-alkyne cycloaddition (SPAAC) has been used for the radiofluorination of bombesin derivatives, RGD peptides, nanobodies and monoclonal antibodies^203,211–216^. Despite its widespread use, the SPAAC ligation is limited by the formation of a lipophilic and bulky benzocyclooctatriazole footprint (Fig. 9b), which can lead to substantial changes in tracer pharmacokinetics^217^. In contrast, the traceless Staudinger ligation offers a useful alternative with a smaller and less lipophilic footprint and has been employed for the radiofluorination of several peptides^218–222^. Major limitations of the Staudinger ligation, however, encompass the lack of in vivo selectivity as well as the slow reaction kinetics at ambient temperature, which confines its utility to in vitro applications involving temperature-resistant vectors^223,224^.

Similar to the SPAAC and Staudinger ligations, the inverse electron demand Diels-Alder (IEDDA) cycloaddition does not require the use of a metal catalyst. The IEDDA reaction proceeds remarkably fast and orthogonal between tetrazine moieties and dienophiles—typically being either *trans*-cyclooctene or norbornene^225^. Given the fast reaction rate, IEDDA cycloadditions are particularly useful for labeling short-lived radionuclides^226^. Along this line of reasoning, this reaction has been used to label a number of peptides, including RGD, Exendin, glucagon-like peptide, and a PARP1 inhibitor analog^227–230^. Nonetheless, it should be noted that isomerization issues, as well as a lipophilic footprint, constitute drawbacks of the IEDDA. While the IEDDA has recently been translated to clinical testing by assessing its utility for drug delivery optimization of doxorubicin (NCT04106492), applications in combination with radiolabeled compounds are eagerly awaited.

#### Pre-targeting approaches

Although antibodies are highly promising vectors due to their excellent affinity and selectivity, they typically exhibit slow pharmacokinetics and biological half-lives of several days. Accordingly, they are only compatible with long-lived radionuclides. Of note, the sluggish clearance from blood circulation, alongside the slow radioactive decay, is associated with high background noise and radiation dose for the patient^231^. To circumvent this core limitation of radiolabeled antibodies, in vivo pre-targeting strategies were developed, whereby a functionalized antibody is injected some days prior to the radionuclide-bearing prosthetic group. Notably, the uncoupling of antibody and radionuclide injection permits the antibody vector to accumulate at the target site, while being cleared from the blood circulation, not only reducing radiation exposure to non-target organs but also improving signal-to-background ratios in regions of interest^176,232^. Once the antibody has largely been cleared from the blood circulation, the radionuclide-bearing prosthetic group is administered, thus allowing the bioconjugation reaction to occur in vivo^233,234^. In addition to the dosimetric benefit to non-target organs and reduced background uptake, pre-targeting with fast and efficient click-type chemistry has rendered antibodies compatible with the labeling with short-lived PET nuclides, including fluorine-18^235–238^. Over the past decade, the IEDDA cycloaddition has proven particularly useful for pretargeting applications^236,239–256^. While there are ongoing efforts to improve the pre-targeting approaches, including fine-tuning between fast kinetics and stability of the reaction partners^257^, preclinical data strongly supports the use of these innovative pre-targeting approaches in humans, and a number of laboratories are currently devoting strenuous efforts toward bringing this exciting concept from bench to bedside^258^.

### Conclusion and perspectives

Recent breakthroughs in molecular imaging with PET have undoubtedly improved diagnostic procedures in clinical oncology, cardiology, and neurology. PET probes are increasingly employed for target engagement studies, thereby facilitating drug discovery and development. However, the rapid growth in nuclear medicine and drug discovery for radiopharmaceuticals poses inherent challenges to radiochemists, since the radiolabeling of many biologically relevant compounds remains difficult. As such, strenuous efforts have been devoted to the development of new radiosynthetic strategies, including the progress from the patents^259–262^. Over the past decade, these efforts provided a plethora of novel synthetic options and broadened the scope of functional groups that can be harnessed for radiolabeling. To date, the most advanced radiofluorination methods, allowing the labeling of both activated and non-activated arenes, include copper-mediated reactions with boron/tin precursors and metal-free hypervalent iodine(III) methods. Similarly, ^11^C-labeling through [^11^C]CO_2_ fixation-mediated carbonylation has been translated to human use and enabled the labeling of various functionalities, including carboxylic acids, amides, carbamates, oxazolidinones, and unsymmetrical ureas. With regard to the labeling of biomolecules, traditional bioconjugation methods that involve the functionalization of primary amines and thiols with NHS, isothiocyanates, anhydrides, or maleimides, respectively, are being well complemented by modern click-type bioconjugation methods such as azide-alkyne cycloadditions and inverse electron demand Diels-Alder reactions. The fast and high-yielding orthogonal radiochemistry with non-naturally occurring chemical functionalities makes the click-type bioconjugations methods particularly promising for future clinical applications. Further, concepts that promote in-house preparation of radiopharmaceuticals have been playing a key role in fostering innovation and enabling cutting-edge applications for patients in need^263^. It is worth mentioning that, attributed to the rapid evolution of PET chemistry, it is critical to follow the consensus nomenclature rules and good practice^33^ in reporting radiochemistry discoveries, facilitating the comparison and translation of novel methods. Indeed, the remarkable advances in radiochemistry have led to a paradigm shift by enabling the design and synthesis of novel radiopharmaceuticals, thereby harnessing innovative concepts and promising approaches to radiotheranostics. In times when precision medicine has reshaped our healthcare system, exploiting such readily available concepts seems invaluable to improve patient management and therapeutic outcomes.
